# A novel pyroptosis-associated lncRNA LINC01133 promotes pancreatic adenocarcinoma development via miR-30b-5p/SIRT1 axis

**DOI:** 10.1007/s13402-023-00818-5

**Published:** 2023-05-04

**Authors:** Jingwei Li, Jiewei Lin, Yuchen Ji, Xuelong Wang, Da Fu, Weishen Wang, Baiyong Shen

**Affiliations:** 1 Department of General Surgery, Pancreatic Disease Center, Ruijin Hospital, Shanghai Jiao Tong University School of Medicine, Shanghai, China; 2 Research Institute of Pancreatic Diseases, Shanghai Jiao Tong University School of Medicine, Shanghai, China; 3 State Key Laboratory of Oncogenes and Related Genes, Shanghai, China; 4 Institute of Translational Medicine, Shanghai Jiao Tong University, Shanghai, China; 5 Department of Thoracic Surgery, Shanghai Pulmonary Hospital, Tongji University School of Medicine, Shanghai, China

**Keywords:** Pyroptosis, Pancreatic adenocarcinoma, Pyroptosis-related lncRNAs, LINC01133, miR-30b-5p

## Abstract

**Purpose:**

Pancreatic adenocarcinoma (PAAD) remains a highly aggressive gastrointestinal malignancy with a dismal prognosis. Pyroptosis has a key role in tumor development. Long noncoding RNAs (lncRNAs) are involved in tumorigenesis and pyroptosis regulation. However, the prognostic potential and function of pyroptosis-related lncRNAs (PRLs) in PAAD remain unclear. We aimed to identify PRLs with promising predictive value for PAAD prognosis and investigate the mechanism by which PRLs affect pyroptosis and PAAD development.

**Methods:**

Key genes that regulate pyroptosis were determined from previous studies, and PRLs were identified from lncRNAs shown to be co-expressed in The Cancer Genome Atlas. Cox analysis and the least absolute shrinkage and selection operator (LASSO) regression model was used to establish a prognostic PRL signature. The clinical significance and functional mechanisms of LINC01133 were explored in vitro and in vivo.

**Results:**

A seven-lncRNA signature was established and the high-risk subgroup exhibited a shorter survival time. With lower immune infiltration abundance, poor immune function, and higher tumor mutational burden (TMB), the high-risk subgroup reflected a more immunosuppressive status with a greater scope for benefiting from immunotherapy. After LINC01133 knockdown, PAAD cells showed lower viability and higher pyroptosis-related gene expression. LINC01133 functioned as a competing endogenous RNA to sequester miR-30b-5p from sponging SIRT1 mRNA to inhibit PAAD pyroptosis.

**Conclusion:**

With significant prognostic value, our PRL signature are involved in the biological processes of PAAD cells and associated with the immune environment. LINC01133 suppresses pyroptosis to promote PAAD development and could serve as a potential target for PAAD treatment.

**Supplementary Information:**

The online version contains supplementary material available at 10.1007/s13402-023-00818-5.

## Background

Pancreatic adenocarcinoma (PAAD) is a highly aggressive gastrointestinal malignancy with a poor prognosis. Given the lack of specific and conspicuous symptoms during the early stages, most patients are not diagnosed until the intermediate and advanced stages; consequently, they may not receive effective treatments. Current tumor markers, such as cancer antigen (CA) 19–9, are insufficient for early PAAD detection or prognosis prediction [1]. With a mean sensitivity of 79% and specificity of 82% for PAAD [2], preoperative CA 19–9 is associated with PAAD and high levels of postoperative CA 19–9 indicate worse outcomes for patients with PAAD [3]. However, owing to its very low positive predictive value, CA 19–9 has limited utility as a screening marker [4]. Recently, genomic profiling studies, particularly multi-omics sequencing and single-cell sequence analysis, have revealed heterogeneity in PAAD and helped to classify patients into different subgroups, thereby improving the feasibility of neoadjuvant therapy, targeted therapy, and immunotherapy [5-8]. Basic bioinformatics-based research on the physiological and pathological processes of PAAD cells can help identify emerging treatment options. Pyroptosis, which has attracted increasing attention in tumor research, is a specific type of programmed cell death induced by various stimuli, such as viruses, toxins, bacteria, and drugs [9]. Pyroptosis has received considerable attention because of its key role in innate immunity and various diseases. Recently, substantial evidence has indicated that it can affect the initiation, development, and prognosis of tumors. The gasdermin (GSDM-) family facilitates pore formation in the cell membrane, leading to the release of inflammatory factors, such as interleukin-18, via cell lysis and inflammation [10, 11]. Pyroptosis can lead to the death of tumor cells, and its pro-inflammatory features affect the tumor microenvironment and tumor immunity [12-14]. For example, in breast cancer, GSDME acts as a tumor suppressor molecule and promotes tumor cell pyroptosis, enhances the phagocytic ability of macrophages, and increases infiltration by CD8 + T and natural killer cells, thereby promoting antitumor immunity [15]. Furthermore, programmed death ligand 1 (PD-L1) can activate GSDMC, thereby promoting cell pyroptosis and enhancing tumor necrosis [16]. Thus, pyroptosis is crucially involved in the development and treatment of tumors, and research on its regulatory mechanism has also received considerable attention in recent years [17].

Long noncoding RNAs (lncRNAs) are critically involved in various tumor bioactivities, such as proliferation, migration, and drug resistance, and some lncRNAs, such as MALAT1, AFAP-AS1, and UCA1, could prompt the diagnosis and prognosis of PAAD [18, 19]. Without encoding proteins, lncRNAs function as key regulators that regulate gene expression in pre-transcriptional, transcriptional, and post-transcriptional processes [20]. The relationship between lncRNAs and pyroptosis has been demonstrated in various diseases and tumors [21, 22]. Wan et al. demonstrated that lncRNA H19 promotes the pyroptosis of retinal neurons in ischemia/reperfusion injury by sponging miR-21, which results in sterile inflammation and neuron damage [23]. Tan et al. found that by sponging the miR-148a-3p, lncRNA HOTTIP regulates the expression of AKT2 to inhibit cell pyroptosis, thereby promoting ovarian cancer development [24]. These findings suggest that lncRNAs regulate pyroptosis, and the study of pyroptosis-related lncRNAs (PRLs) can reveal the pathophysiological mechanisms of tumors. Furthermore, the clinical significance of PRLs and their detailed functional mechanism in PAAD cells have not been clearly determined and require further investigation.

LINC01133 plays a key role in various tumor types, such as lung squamous cell carcinoma, colorectal cancer, and PAAD [25]. Furthermore, LINC01133 regulates the epithelial-mesenchymal transition of PAAD via the Wnt/β-catenin pathway and promotes tumorigenesis via the upregulation of CCNG1 [26]. However, little is known about the relationship between LINC01133 and pyroptosis. Therefore, we aimed to identify PRLs with promising predictive value for PAAD prognosis, investigate the mechanism by which PRLs affect pyroptosis and PAAD development and finally find the potential novel target for PAAD.

## Methods

### Data source

Transcriptome sequences and clinical information of patients with PAAD were downloaded from The Cancer Genome Atlas (TCGA; https://portal.gdc.cancer.gov/) databases. Owing to a lack of sufficient follow-up (less than 30 days) and transcriptome expression data, six TCGA samples were excluded, and a total training cohort of 171 patients from TCGA was included in our study. Patients in the Ruijin Hospital PAAD cohort were used as the testing cohort. Somatic mutation information was obtained from TCGA. The immune infiltration abundance data of TCGA patients were derived from the TIMER2.0 website (http://timer.cistrome.org/) [27]. The microarray data were obtained from the Gene Expression Omnibus (GEO; http://www.ncbi.nlm.nih.gov/geo/), and we included GSE16515 and GSE32676 for further analysis.

### Identification of PRLs

The pyroptosis-related gene list (see Supplementary Table 1, Online Resource 1), which contains 55 genes, was obtained from MSigDB (version 7.4, REACTOME_PYROPTOSIS) and existing literature [10, 28, 29]. The PRLs were identified using Pearson's correlation analysis with the “limma” R package in the training cohort with R^2^ > 0.5 and *p* < 0.001.

### Establishment of a pyroptosis risk-related lncRNA signature with prognostic value

First, the differentially expressed lncRNAs in the pyroptosis-related lncRNA list were screened using the “limma” R package (P-value < 0.05 and |Log2FC|> 1). Next, based on the list therein of differentially expressed PRLs, a univariate Cox screen analysis was used to identify prognostic PRLs with *p* < 0.01. Thereafter, a least absolute shrinkage and selection operator regression model was established using the “glmnet” package. Finally, multivariate Cox analysis was performed to obtain the PRL prognostic signature for patients with PAAD. After calculating the risk scores, the patients were divided into risk subgroups based on suitable cut-off points. The Ruijin PAAD cohort was used to verify the prognostic value of the PRL signature.

### Assessment of prognostic factors

TCGA-PAAD patients were clustered into subgroups related to their clinical characteristics and Kaplan–Meier (KM) survival analysis was performed for each subgroup of the different risk groups. Univariate and multivariate Cox regression analyses of clinical features and risk scores were performed to identify independent prognostic factors. A nomogram was constructed to predict 12-, 24-, and 36-month survival probabilities for patients with PAAD.

### Gene set enrichment analysis

The differentially expressed genes (DEGs) between the different risk subgroups were obtained and used for Gene Ontology (GO) [30] and Kyoto Encyclopedia of Genes and Genomes (KEGG) [31] analyses. Gene Set Enrichment Analysis (GSEA; versions c5.go.v7.4 symbols.gmt and c2.cp.kegg.v7.4. symbols.gmt) was performed to explore the potential function of PRLs.

### Mutation analysis

Mutation data from TCGA were downloaded to identify the differences between the risk subgroups. The “maftools” R package was used to identify genes with high mutation frequency; these genes were illustrated using waterfall plots. The correlation between the tumor mutation burden (TMB) and our prognostic risk signature was analyzed.

### Immune landscape description

We executed the Cell‐type Identification By Estimating Relative Subsets of RNA Transcripts (CIBERSORT) algorithm to calculate the immune cell infiltration abundance of the different risk subgroups. The expression of common checkpoints was also assessed. The correlation between PRLs and immune cells was analyzed using the “preprocessCore” R package.

### Chemotherapy drug response analysis

The IC50 values of common chemotherapeutic drugs for the different risk subgroups were calculated using the “pRRophetic” R package to assess the implication of our signature for clinical treatment.

### Clinical samples

Seventy-one human PAAD tissues and adjacent normal tissues were obtained from patients that did not receive any preoperative chemotherapy in the Ruijin Hospital affiliated with the Shanghai Jiaotong University School of Medicine (Shanghai, China). Tissues were stored at − 80 °C until use. The protocols were approved by the Institutional Ethics Committee of Ruijin Hospital and all participants provided informed consent.

### Cell culture and transfection

PAAD cell lines (BXPC-3, CFPAC-1, MIA-PACA-2, PANC-1, and PATU-8988), human normal pancreatic ductal epithelial cells (HPNE), and HEK293T cells were obtained from the Cell Bank of the Chinese Academy of Sciences (Shanghai, China). The cells were routinely tested for mycoplasma and cultured in RPMI-1640, Iscove's modified Dulbecco's medium, and Dulbecco's modified Eagle medium supplemented with 10% fetal bovine serum.

For in vitro experiments, si-LINC01133#1, si-LINC01133#2, si-NC, si-SIRT1#1, miR-30b-5p mimics, and miR-30b-5p inhibitor were obtained by BioeGene (Shanghai, China). After transfection with Lipofectamine 3000 for 2 days, cells were harvested for subsequent experiments. The small hairpin RNA (shRNA) of LINC01133 was cloned into the piLenti-shRNA vector by BioeGene. HEK293T cells were used to produce the corresponding lentivirus, which was then transduced into PANC-1 and PATU-8988 cells. The PAAD cells were treated with puromycin for 2 days to obtain stable LINC01133 knockdown cell lines. The siRNA, miRNA mimic, and shRNA information is available in Supplementary Table 2, 3, and 6, Online Resource 1.

### Quantitative real-time PCR

TRIzol (Invitrogen, Carlsbad, CA, USA) was used to extract total RNA from pancreatic tissues and cell lines. The PrimeScript RT Reagent Kit (TaKaRA Bio Inc., Shiga, Japan) was used for reverse transcription, according to the manufacturer’s instructions. Applied Biosystems Fast SYBR Green Master Mix (Thermo Fisher Scientific, Waltham, MA, USA) was used for the quantitative real-time PCR (qRT-PCR) assay. ACTB and U6 were employed as mRNA and miRNA internal controls, respectively. A list of primer sequences used in this study is provided in Supplementary Table 4, Online Resource 1.

### Western blotting

Cells were harvested and lysed using RIPA buffer (Thermo Fisher Scientific) mixed with protease inhibitors. The proteins were then loaded on a 4–20% SDS-PAGE gel (GenScript, Nanjing, China). Separated proteins were transferred onto PVDF membranes and the membranes were incubated using the following primary antibodies: anti-caspase 1 (1:1000; 43811; Cell Signaling Technology [CST], Danvers, MA, USA), anti-cleaved caspase 1 (1:1000; 43811; CST), anti-GSDMC (1:1000; 43811; CST), anti-NRLP3 (1:1000; 38679; SAB), anti-ASC (1:1000; 10500–1-AP; Proteintech, Rosemont, IL, USA), anti-β-Actin (1:1000; 3700; CST), and anti-SIRT1 (1:1000; 13161–1-AP; Proteintech). After incubation with the corresponding secondary antibody, protein expression was detected using the enhanced chemiluminescence reagent. β-Actin was used as an endogenous control.

### RNA fluorescence *in situ* hybridization analysis

For the fluorescence in situ hybridization (FISH) assay, the Ribo LINC01133 Probe Mix was synthesized and used to investigate the localization of LINC01133 in PAAD cells. The detailed protocol was previously described [32]. The FISH probe information is available in Supplementary Table 5, Online Resource 1.

### Immunohistochemical and *in situ* hybridization analyses

Digoxigenin-labeled LINC01133 probes were obtained from Servicebio (Wuhan, China). Immunohistochemical (IHC) and in situ hybridization (ISH) staining were conducted using PAAD tissues and adjacent normal tissues from patients and xenograft tumor mice, as previously described [32]. Cleaved-GSDMD, TUNEL, SIRT1, and LINC01133 staining scores were calculated by multiplying the positive scores, which were based on the proportion of positively stained cells and ranged from 0 to 4 (represented as 0%, 1–25%, 26–50%, 51–75%, and 76–100%, respectively), and intensity scores [scored as 0 (absent), 1 (weak), 2 (moderate), or 3 (strong)].

### Flow cytometric analysis

After transfection for 48 h, 1.5 × 10^5^ PAAD cells were incubated with fluorescein isothiocyanate-conjugated annexin V and propidium iodide for 30 min in the dark at room temperature and analyzed using flow cytometry.

### Cell viability assay

Cell viability was assessed using a CCK-8 (Cell Counting Kit 8, Abcam, Cambridge, UK) assay. After transfection for 48 h, PAAD cells (1.5 × 10^3^ per well) were harvested and cultured in a 96-well plate for an additional 5 days. Then, 10 μL of CCK-8 test reagent was added each day and the number of living cells was measured by absorbance at 460 nm after 2 h.

### TUNEL assay

For the TUNEL assay, transfected PAAD cells (1.5 × 10^5^ per well) were plated in 6-well plates for 2 days and then fixed and stained using the TUNEL reagent kit (Beyotime, Shanghai, China), according to the manufacturer’s instructions. The ratio of red- to blue-colored cells represented the ratio of TUNEL-positive cells.

### Dual-luciferase reporter assay

The StarBase 3.0 (http://starbase.sysu.edu.cn) database was used to predict the direct interaction of LINC01133 and miR-30b-5p. The binding site of miR-30b-5p with SIRT1 was predicted using RAID, miRDB, and TargetScan databases. According to the binding site, wide-type and mutant sequences were synthesized and inserted into a pGL3-basic luciferase reporter vector. HEK293T cells were co-transfected with the vectors with miR-30b-5p mimic or negative controls (BioeGene). The dual-luciferase reporter assay system was used to determine luciferase activity (Promega, Madison, WI, USA).

### Tumor xenograft assay

Female BALB/c nude mice (6 weeks old) were obtained from the Chinese Academy of Sciences. LINC01133-stable knockdown and negative-control PAAD cells were subcutaneously injected into mice (3 × 10^5^ per mouse, n = 7 per group). Every 4 days following the injection, the largest length (L) and width (W) were measured to calculate the tumor volumes as follows: volume = 1/2 (L × W^2^). Thirty-two days after the initial injection, the subcutaneous tumors were separated from the mice and their weights were measured. The xenograft tumors were then fixed using formalin and used for hematoxylin–eosin (HE) or IHC staining.

### Statistical analyses

R software (version 4.1.0) was used for statistical analyses and plots. Transcriptome and clinical data were merged using PERL (version 5.30.2; http://www.perl.org/). Experimental results are shown as the mean ± standard deviation (SD). To compare the means of the various groups, one-way analysis of variance, Student's *t*-test, and Chi-square test were performed. The KM method was employed to construct survival curves, and log-rank tests were performed to assess group differences. The cut-off value for statistical significance was *p* < 0.05.

## Results

### Identification of PRLs

We downloaded sequencing data and clinical and mutation information for 182 samples from TCGA-PAAD database (179 tumors and 4 normal samples) and found 14,041 lncRNAs in the sequencing data. Then, 1,208 lncRNAs were found to be co-expressed with key regulator genes for pyroptosis with R^2^ > 0.5 and *p* < 0.001. PRL expression in cancer and normal tissues was calculated, and 80 differentially expressed lncRNAs were obtained for subsequent analysis (*p* < 0.05, |Log2FC|> 1). See Supplementary Fig. 1, Online Resource 2 for a flow diagram of our methods.

### Construction of the prognostic PRL signature

**Fig. 1:**
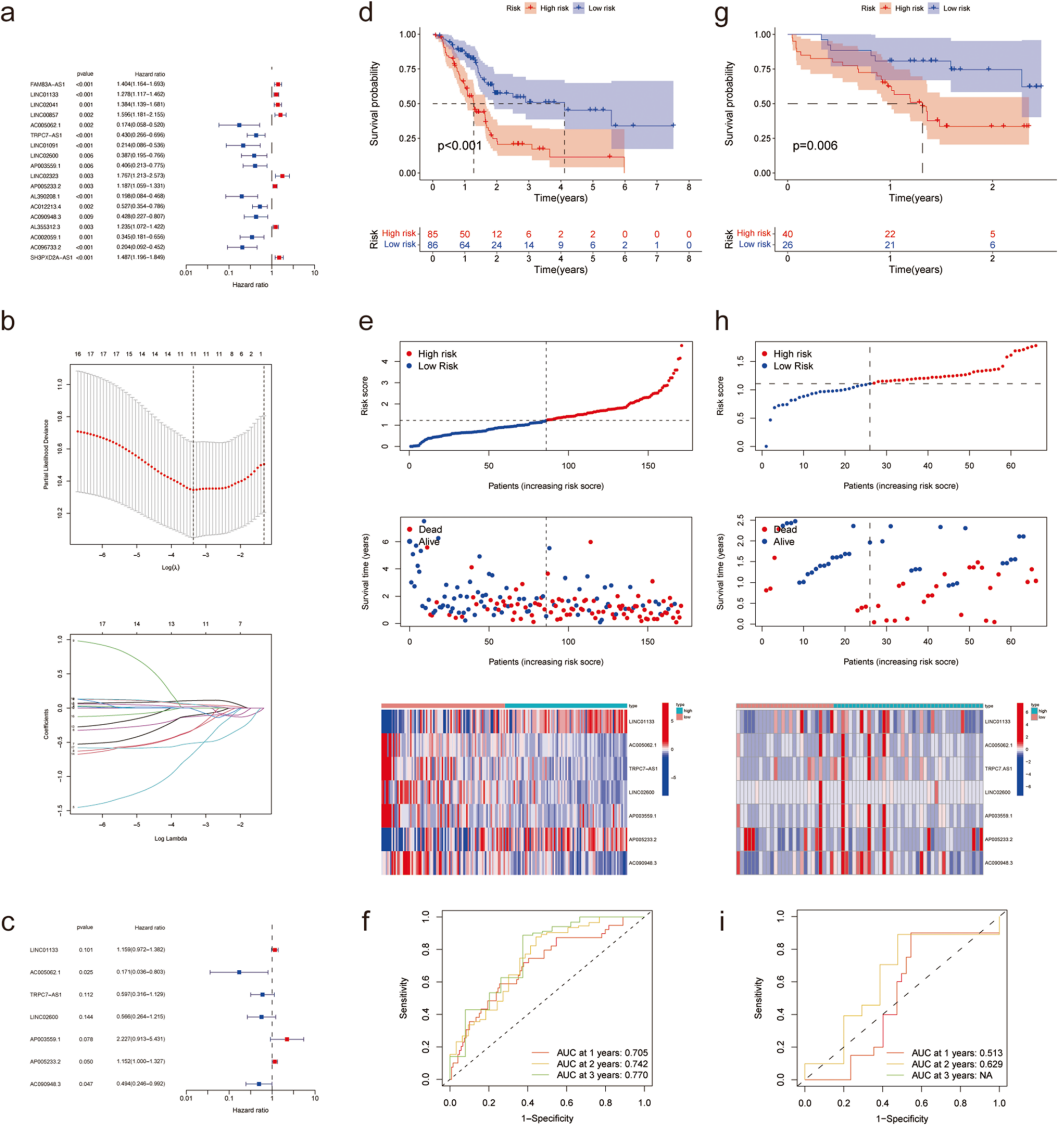
Selection of PRLs and validation of the prognostic value of the PRL signature. (**a**) Univariate Cox regression showing 18 PRLs correlated with OS in TCGA-PAAD. (**b**) LASSO logistic regression algorithm to screen PRLs associated with prognosis in PAAD**.** (**c**) Multivariate Cox regression results showed a PRL signature including seven lncRNA that is associated with prognosis in PAAD. (**d, g**) KM survival curves show the OS of patients in the high- and low-risk subgroups in TCGA-PAAD database (**d**) and Ruijin database (**g**). (**e, h**) Risk score distribution plots, survival distribution plots, and PRL signature expression plots from TCGA-PAAD database (**e**) and Ruijin database (**h**). (**f, i**) Time‐dependent ROC curves based on the PRL signature at 1 year in TCGA-PAAD database (**f**) and Ruijin database (**i**)

Univariate Cox regression analysis was performed for TCGA-PAAD cohort (using the clinical and sequencing data of 171 patients) to assess the prognostic relevance of the 80 differentially expressed lncRNAs, which found 18 lncRNAs that correlated with patient prognoses (*p* < 0.01) (Fig. 1a). Next, to further reduce the range of potential PRLs, we established the LASSO regression model and determined a list of 11 lncRNAs (Fig. 1b) that were used for further multivariate Cox regression analysis (Fig. 1c). Finally, we established a predictive PRL signature including seven PRLs as follows: risk score = (0.1474 × expression level of LINC01133) – (1.7668 × expression level of AC005062.1) – (0.5157 × expression level of TRPC7-AS1) – (0.569 × expression level of LINC02600) + (0.8005 × expression level of AP003559.1) + (0.1418 × expression level of AP005233.2) – (0.7057 × expression level of AC090948.3) (Table 1). Based on their risk scores, the patients were divided into different risk subgroups. The high-risk subgroup showed a significantly lower overall survival (OS) time than the low-risk subgroup (Fig. 1d, e). Moreover, the area under the curve (AUC) values of receiver operating characteristic (ROC) for 1-, 2-, and 3-year OS were 0.705, 0.742, and 0.770, respectively (Fig. 1f). Overall, the results demonstrated the high predictive value of the PRL risk signature.

**Table 1:** Multivariate Cox results for PRLs based on TCGA-PAAD

ID	Coef.	HR	HR.95L	HR.95H	*P*-value
LINC01133	0.1474	1.1589	0.9716	1.3822	0.101
AC005062.1	–1.7668	0.1709	0.0364	0.803	0.0252
TRPC7-AS1	–0.5157	0.5971	0.3159	1.1286	0.1124
LINC02600	–0.569	0.5661	0.2637	1.2152	0.1443
AP003559.1	0.8005	2.2267	0.9129	5.4312	0.0785
AP005233.2	0.1418	1.1523	1.0003	1.3274	0.0495
AC090948.3	–0.7057	0.4938	0.2459	0.9916	0.0473

*Coef.* regression coefficient; *HR* hazard ratio

### Validation of the PRL signature

We used the Ruijin Hospital PAAD database as a validation cohort to verify the predictive value of our PRL signature for the prognosis of patients with PAAD. After calculating the risk score, patients were classified into high- and low-risk subgroups based on the cut-off points. KM survival analysis showed that our risk score was an important predictor of OS (Fig. 1g, h). As the AUC values of ROC for 1- and 2-year OS were greater than 0.5, the reliability of our signature was verified (Fig. 1i). Similarly, across all TCGA subgroups classified by clinical features, including age, sex, T stage, grade, and the American Joint Committee on Cancer (AJCC) stage, with lower OSs, patients in the high-risk subgroup showed a significantly worse prognosis than those in the low-risk subgroup (See Supplementary Fig. 2, Online Resource 2).

### Clinical value and function analysis of the PRL signature

**Fig. 2:**
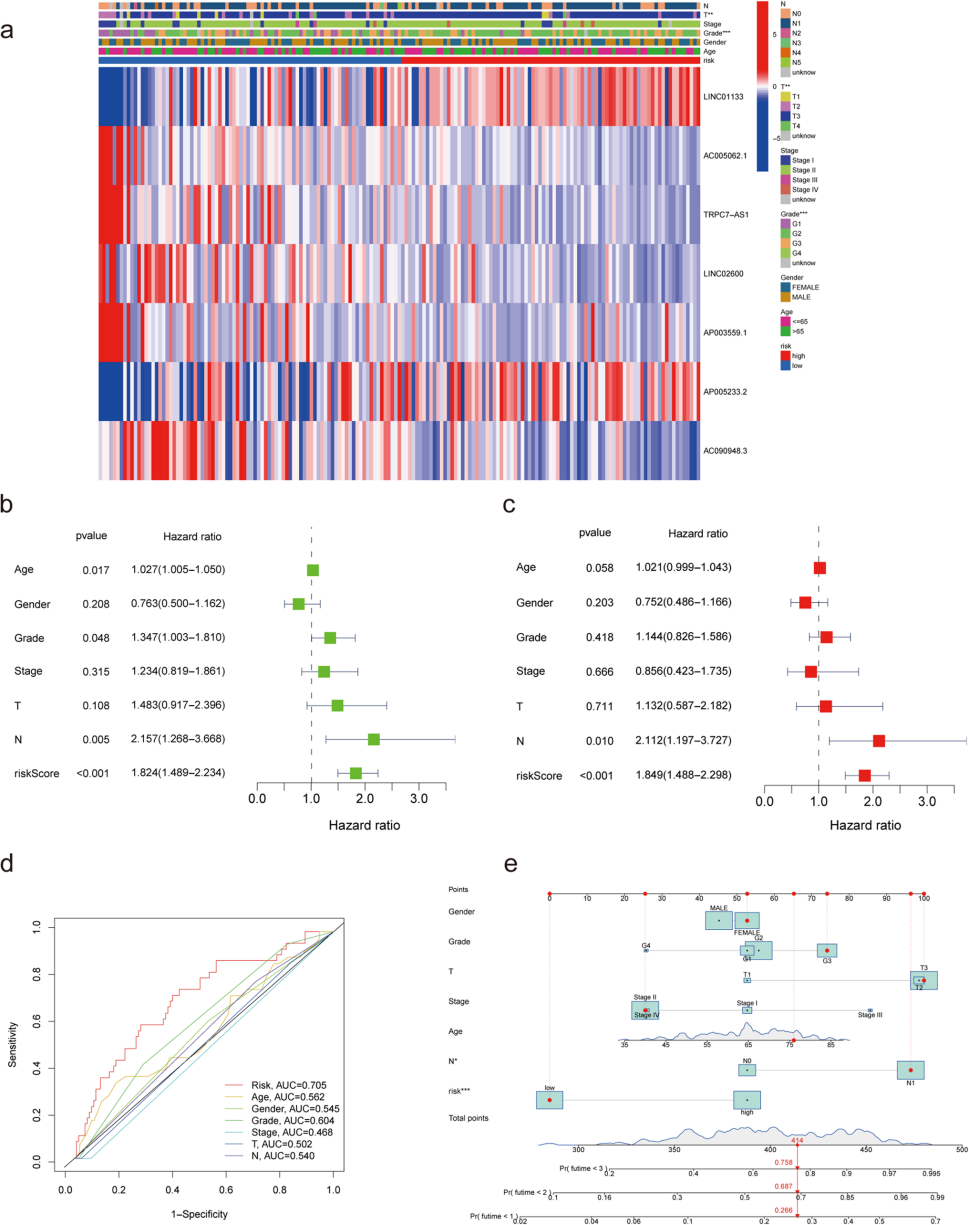
Estimated prognostic accuracy of the PRL signature in patients with PAAD. (**a**) The connection between the risk score with the clinical characteristics. (**b**) Univariate Cox regression indicating that the age, grade, N stage, and risk score were correlated with OS (*p* < 0.05). (**c**) Multivariate Cox regression showing that the N stage and the risk score (*p* < 0.05) were independent prognostic factors of OS in patients with PAAD. (**d**) ROC curve showing that the risk score has the highest prognostic accuracy. (**e**) Prognostic nomogram based on the risk score using the prognostic PRL signature and the clinical features to predict 1-, 2-, and 3-year survival in patients with PAAD (*p < 0.05; **p < 0.01; ***p < 0.001; ns, no significance)

In TCGA database, patients in the high-risk subgroup showed higher T stages and grades than those in the low-risk subgroup, which was consistent with a higher degree of malignancy (Fig. 2a, Table 2). Cox regression analysis was performed to compare the prognosis prediction value of our signature with other commonly used clinical markers. Univariate Cox regression analysis suggested that age, histological grade, N stage, and risk score significantly correlated with prognosis in patients with PAAD (Fig. 2b). When clinical features were controlled, the multivariate Cox regression results demonstrated that the risk score was still an independent prognosis-related factor in patients with PAAD (*p* < 0.001). However, age and histological grade were no longer associated with prognosis in the multivariate regression analysis (Fig. 2c). The risk score for our PRL signature showed a significantly higher AUC value of the ROC curve than that for other clinical features (Fig. 2d), indicating a better predictive ability for prognosis. As a common prognostic visualization tool, nomograms were constructed using the factors obtained from multivariate regression to predict the 1-, 2-, and 3-year survival probabilities of patients with PAAD. Figure 2e shows the nomogram results for one patient in TCGA-PAAD cohort. Collectively, these results indicate that the PRL signature could serve as a promising tool to predict prognosis in patients with PAAD, as a lower risk score indicates a better prognosis.

**Table 2:** Correlation between PRL risk groups and clinical characteristics based on TCGA-PAAD

Clinicopathologic	Case	PRL risk groups		*P*-value
parameters	(*n* = 171)	Low	High	
Total	171	86	85	
Gender				0.999
Male	93	47	46	
Female	78	39	39	
Age				0.622
≥ 60	117	57	60	
< 60	54	29	25	
Grade				< 0.001
G1	28	25	3	
G2	92	36	56	
G3	47	23	24	
G4	2	1	1	
unknown	2	1	1	
Pathological stage				0.088
I	19	14	5	
II	142	67	75	
III-IV	7	3	4	
unknown	3	2	1	
T stage				0.001
T1-2	28	22	6	
T3-4	141	62	79	
unknown	2	2	0	
Lymph node metastasis				0.731
N0	47	25	22	
N1	119	58	61	
unknown	5	3	2	
Distant metastasis				0.617
M0	77	38	39	
M1	4	1	3	
unknown	90	47	43	

**Fig. 3:**
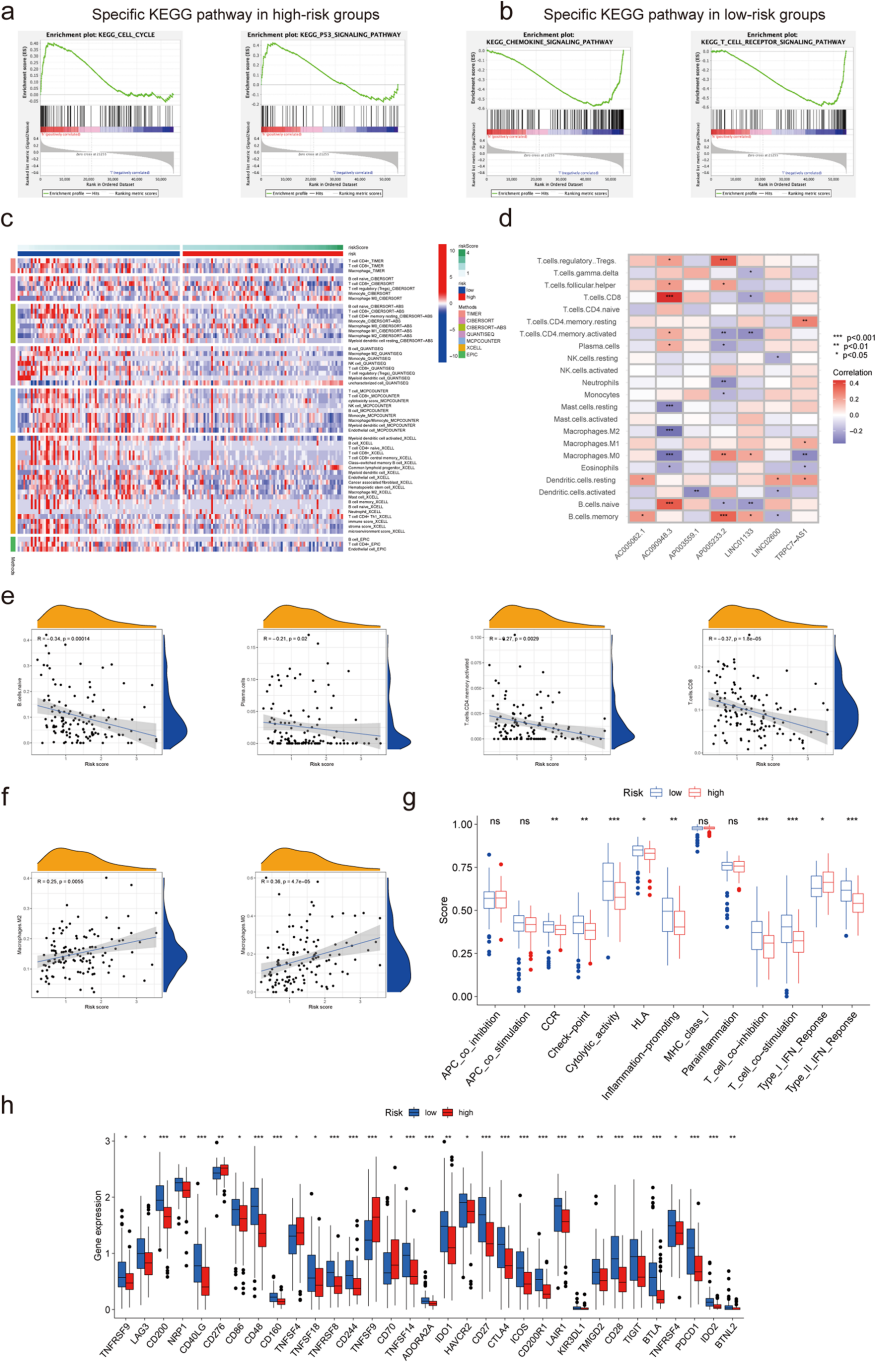
Immune profile of TCGA-PAAD dataset. (**a, b**) GSEA of the PRL signature for KEGG pathways in the two subgroups. (**c**) Heatmap showing immune cell infiltration analysis among the two subgroups. (**d**) Correlation between the seven PRLs and different types of immune cells. (**e**) Correlation between naïve B cell, plasma cell, CD4 + T memory activated cell, and CD8 + T cell infiltration with the PRL signature. (**f**) Correlation between M0 and M2 macrophage type and the PRL signature. (**g**) Immune function enrichment by single-sample GSEA. (**h**) Immune checkpoint expression in the two subgroups (*p < 0.05; **p < 0.01; ***p < 0.001; ns, no significance)

To explore the significant changes of functional phenotypes in high- and low-risk subgroups, 1,717 DEGs in TCGA were subjected to GO and KEGG analyses and the results suggested that the DEGs were mainly associated with cellular interactions and signal transduction (See Supplementary Fig. 3a, b, Online Resource 2). The results of the GSEA–GO analysis indicated that the high-risk subgroup mainly contained genes involved in cell structure processes, whereas the low-risk subgroup mainly contained genes involved in the metabolic processes of cells (See Supplementary Fig. 3c, d, Online Resource 2). In the GSEA–KEGG analysis, cell proliferation-related pathways, such as the “cell cycle” and “p53 signaling pathway,” were enriched in the high-risk subgroup (Fig. 3a). In contrast, the low-risk subgroup contained genes involved in inflammation and tumor immunity processes, such as the “T cell receptor signaling pathway,” “B cell receptor signaling pathway,” and “chemokine signaling pathway” (Fig. 3b).

### Immune landscape

Because the functional enrichment analysis revealed that our PRL signature was correlated with the immune response, we explored the association of our signature with the tumor immune microenvironment. After analyzing the immune cell infiltration data from TCGA from TIMER2.0, we found higher infiltration of B cells and CD8 + T cells in the low-risk subgroup, while M0 macrophages were enriched in the high-risk subgroup (Fig. 3c).

In our PRL signature. AC090948.3 was positively correlated with CD8 + T and naïve B cell infiltration and AP005233.2 was positively associated with regulatory T cells (Tregs) and M0 macrophages (Fig. 3d). The results of the correlation analysis between our risk score and the infiltration of different types of immune cells suggested that the infiltration of naïve B cells, plasma cells, memory CD4 + T cells, and CD8 + T cells was inversely associated with the risk score (Fig. 3e), whereas M0 and M2 macrophage infiltration was positively correlated with the risk score (Fig. 3f). Next, the immune function analysis results suggested a stronger pro-inflammatory and cytolytic function, as well as higher chemokine receptor and immune checkpoint-related functions, in the low-risk subgroup, whereas the high-risk subgroup showed a stronger type I interferon response (Fig. 3g). Thus, these data showed a correlation between our risk score and the immunosuppressive tumor microenvironment.

Finally, we analyzed the expression levels of common immune checkpoint factors in the two subgroups and found that many of them, such as CTLA4 and PDCD1, were highly expressed in the low-risk subgroup, whereas only a few immune checkpoint factors, such as CD276, had elevated expression levels in the high-risk subgroup (Fig. 3h). Elevated levels of LAG3, CTLA4, and PDCD1 indicated the exhaustion of immune cells. Collectively, these results provide information to improve future immunotherapy for PAAD.

### Somatic mutations, stemness score, and therapeutic response

It is known that gene mutations play an important part in PAAD initiation and development. In the high-risk subgroup, the mutation frequencies for most genes, TMB score, and stemness score were higher than in the low-risk subgroup (See Supplementary Fig. 4a–e, Online Resource 2), which suggested a higher degree of malignancy in patients in the high-risk subgroup and that immune checkpoint therapy may contribute to a better outcome for patients in the high-risk subgroup [33]. We then assessed the therapeutic response for commonly used chemotherapy drugs for PAAD in different subgroups [34]. With higher IC50 values for paclitaxel and docetaxel in the low-risk subgroup but no differences in that for gemcitabine, the patients in the high-risk subgroup might be more sensitive to paclitaxel and docetaxel (See Supplementary Fig. 4f-h, Online Resource 2).

### LINC01133 is highly expressed in PAAD tissues and cell lines and associated with a worse outcome

**Fig. 4:**
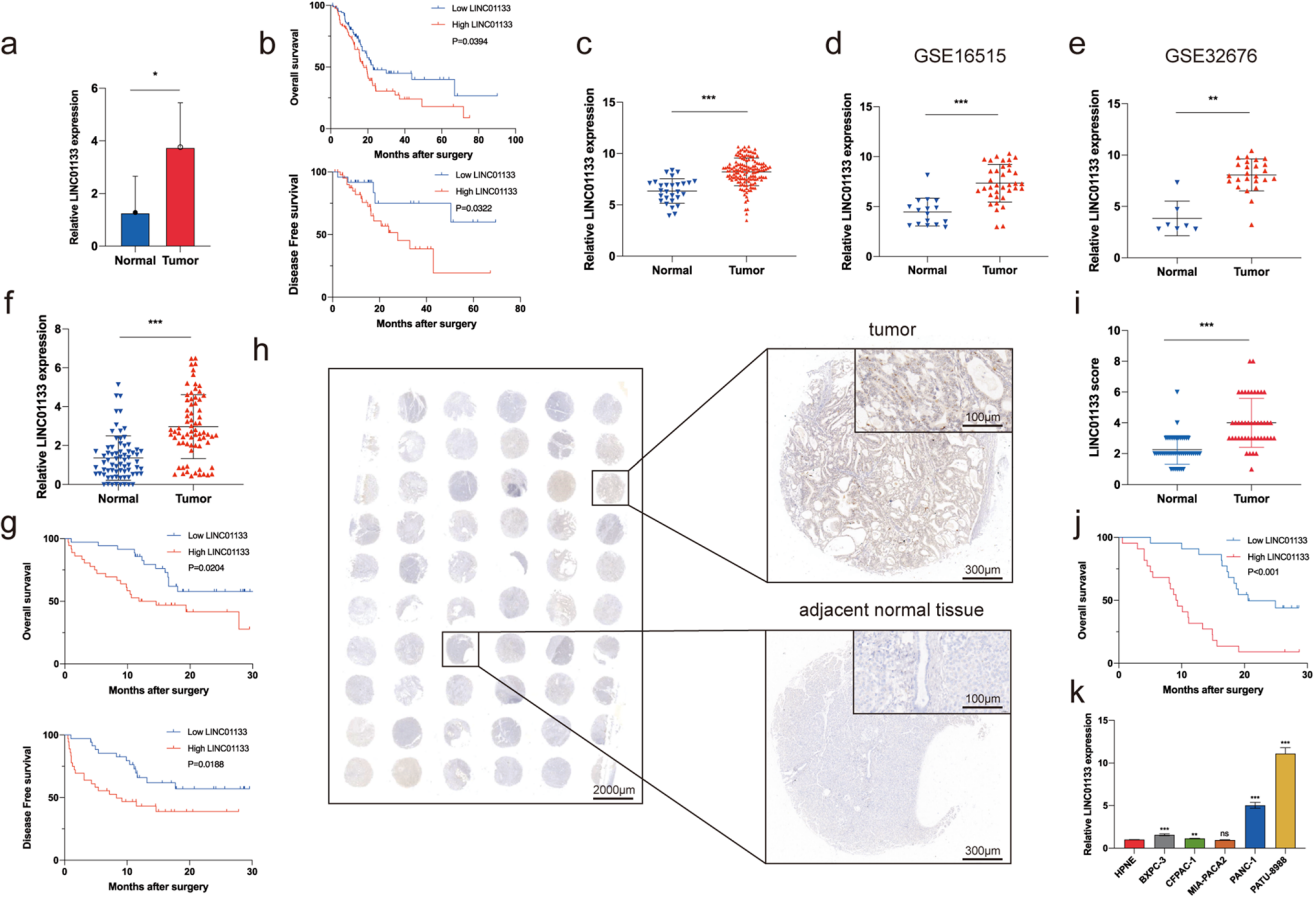
LINC01133 expression is elevated in PAAD tissues and cell lines and correlates with poor prognosis. (**a**) LINC01133 expression in TCGA database. (**b**) Overall survival (OS) and disease-free survival (DFS) based on LINC01133 expression levels were determined using TCGA database. **(c–e)** LINC01133 expression in multi-omics datasets (**c**), GSE16515 (**d**), and GSE32676 (**e**). (**f, g**) LINC01133 expression in 71 pairs of PAAD tumor tissues and adjacent normal tissues from Ruijin Hospital (**f**) is associated with the poor OS and DFS of patients (**g**). (**h**) Representative image of LINC01133 ISH staining. (**i**) LINC01133 ISH staining scores in 44 pairs of PAAD tumor tissues and adjacent normal tissues from our center. (**j**) Prognostic analysis of LINC01133 using clinical prognostic data from 44 patients based on the LINC01133 staining scores. (**k**) LINC01133 expression in PAAD cell lines (BXPC-3, CFPAC-1, MIA-PaCa-2, PANC-1, and PATU-8988) compared with that of normal pancreatic ductal epithelial cell line HPNE detected using qRT-PCR. *p < 0.05; **p < 0.01; ***p < 0.001; ns, no significance

To explore potential therapeutic hub genes in the PRL signature, we examined the expression and prognostic prediction value of single lncRNAs based on TCGA-PAAD database and their relationship with patient prognosis (See Supplementary Fig. 5a–c, Online Resource 2). We found that LINC01133 (Fig. 4a, b) and AP005233.2 were highly expressed in PAAD, and their expression levels were negatively correlated with patient prognosis. However, the expression level of AP005233.2 was not significantly correlated with prognosis in Ruijin-PAAD cohort (See Supplementary Fig. 5d, Online Resource 2). Furthermore, we found that the LINC01133 expression level was associated with a higher grade for PAAD (Table 3). Another multi-omics study (Fig. 4c) [35] and two GEO databases, GSE16515 (Fig. 4d) and GSE32676 (Fig. 4e), demonstrated the overexpression of LINC01133 in PAAD. Moreover, TCGA database indicated that the expression of LINC01133 was upregulated in several other tumors, and GSEA results indicated that LINC01133 was connected to pathways related to PAAD, lung cancer, bladder cancer, and colon cancer (See Supplementary Fig. 6a, b, Online Resource 2). These findings imply that LINC01133 has a significant impact on various tumors, particularly PAAD. To further verify this hypothesis, we used the RNA samples (Fig. 4f, g) and tissue microarrays (Fig. 4h–j) from our center to study the expression of LINC01133 and obtained the same expression and prognosis results as that obtained from TCGA. Finally, we validated LINC01133 expression in cell lines, and the results suggested that LINC01133 was more highly expressed in the four PAAD cell lines than in HPNE cells (Fig. 4k). In conclusion, these findings imply a significant upregulation of LINC01133 in PAAD and that high LINC01133 expression is correlated with a worse prognosis.

**Table 3:** Correlation between LINC01133 expression level and clinical characteristics based on TCGA-PAAD

Clinicopathologic	Case	LINC01133 expression level		*P*-value
parameters	(*n* = 171)	Low	High	
Total	171	86	85	
Gender				0.646
Male	93	45	48	
Female	78	41	37	
Age				0.870
≥ 60	117	58	59	
< 60	54	28	26	
Grade				< 0.001
G1	28	23	5	
G2	92	36	56	
G3	47	24	23	
G4	2	2	0	
unknown	2	1	1	
Pathological stage				0.226
I	19	13	6	
II	142	68	74	
III-IV	7	3	4	
unknown	3	2	1	
T stage				0.107
T1-2	28	18	10	
T3-4	141	66	75	
unknown	2	2	0	
Lymph node metastasis				0.999
N0	47	23	24	
N1	119	60	59	
unknown	5	3	2	
Distant metastasis				0.617
M0	77	38	39	
M1	4	1	3	
unknown	90	47	43	

### LINC01133 inhibits pyroptosis and promotes PAAD cell viability *in vitro* and *in vivo*

**Fig. 5:**
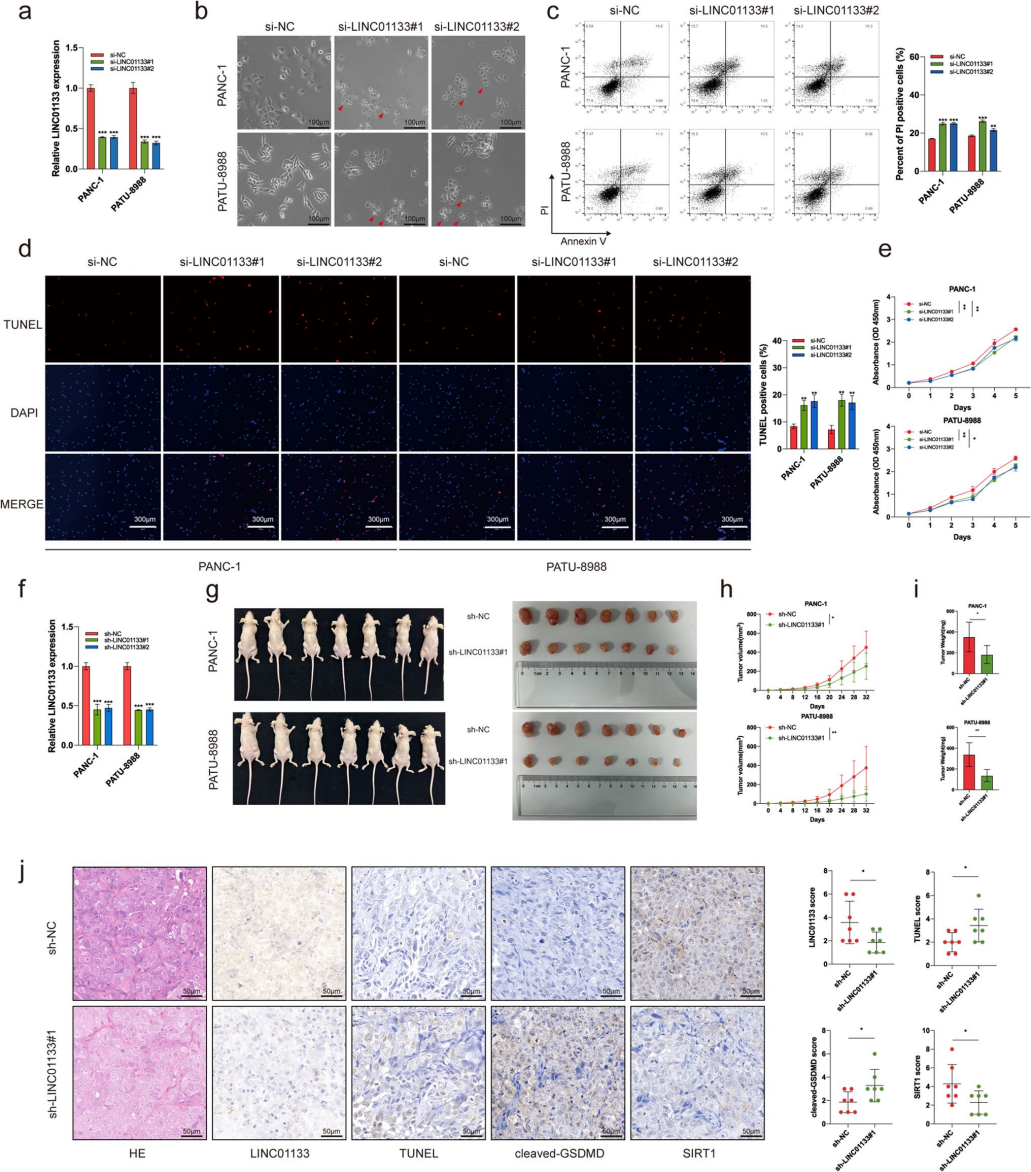
LINC01133 promotes the viability of PAAD cells in vitro and in vivo by inhibiting pyroptosis. (**a**) LINC01133 expression was detected using qRT-PCR after siRNA transfection. (**b**) Representative bright-field image of cell morphology change after siRNA transfection; the red arrow indicate the large bubbles emerging from the plasma membrane. (**c**) Flow cytometry analysis of annexin V/PI-stained PAAD cells. (**d**) TUNEL assays were used to detect cell death after transfection with LINC01133 siRNAs. (**e**) PANC-1 and PATU-8988 cells were subjected to CCK-8 assays after transfection with LINC01133 siRNAs. (**f**) LINC01133 expression was detected using qRT-PCR after lentivirus transduction. (**g–i**) Images (**g**), volume (**h**), and weights (**i**) of subcutaneous tumors in each group. (**j**) Representative photographs of HE, LINC01133 ISH staining, TUNEL, cleaved-GSDMD, and SIRT1IHC staining in xenograft tumors. *p < 0.05; **p < 0.01; ***p < 0.001; ns, no significance. All in vitro experiments were repeated three times

To elucidate the function of LINC01133 in PAAD cell behavior, we transfected si-NC, si-LINC01133#1, and si-LINC01133#2 into PANC-1 and PATU-8988 and detected the LINC01133 expression level using qRT-PCR (Fig. 5a). After knockdown of LINC01133, some PAAD cells exhibited bubbles emerging from the membrane as well as cell swelling (Fig. 5b), which resembled the pyroptosis process. Next, a higher ratio of PI-positive and TUNEL-positive cells was detected in the knockdown group (Fig. 5c, d). In addition, the results of CCK-8 assay indicated that knocked down LINC01133 inhibited PAAD cell viability. (Fig. 5e). These results suggested that LINC01133 might inhibit PAAD pyroptosis and promote cell viability [36]. In vivo experiments verified the results. We found that the knockdown of LINC01133 significantly reduced tumor weight and volume compared with those in the NC group (Fig. 5f–i). IHC results of xenograft tumor tissue implied that the tumors with low LINC01133 expression showed higher TUNEL expression (Fig. 5j). Collectively, these results supported that LINC01133 inhibits the pyropsotis and promotes the viability of PAAD cells in vitro and in vivo.

### LINC01133 ameliorates PAAD pyroptosis *via* the miR-30b-5p/SIRT1 axis

**Fig. 6:**
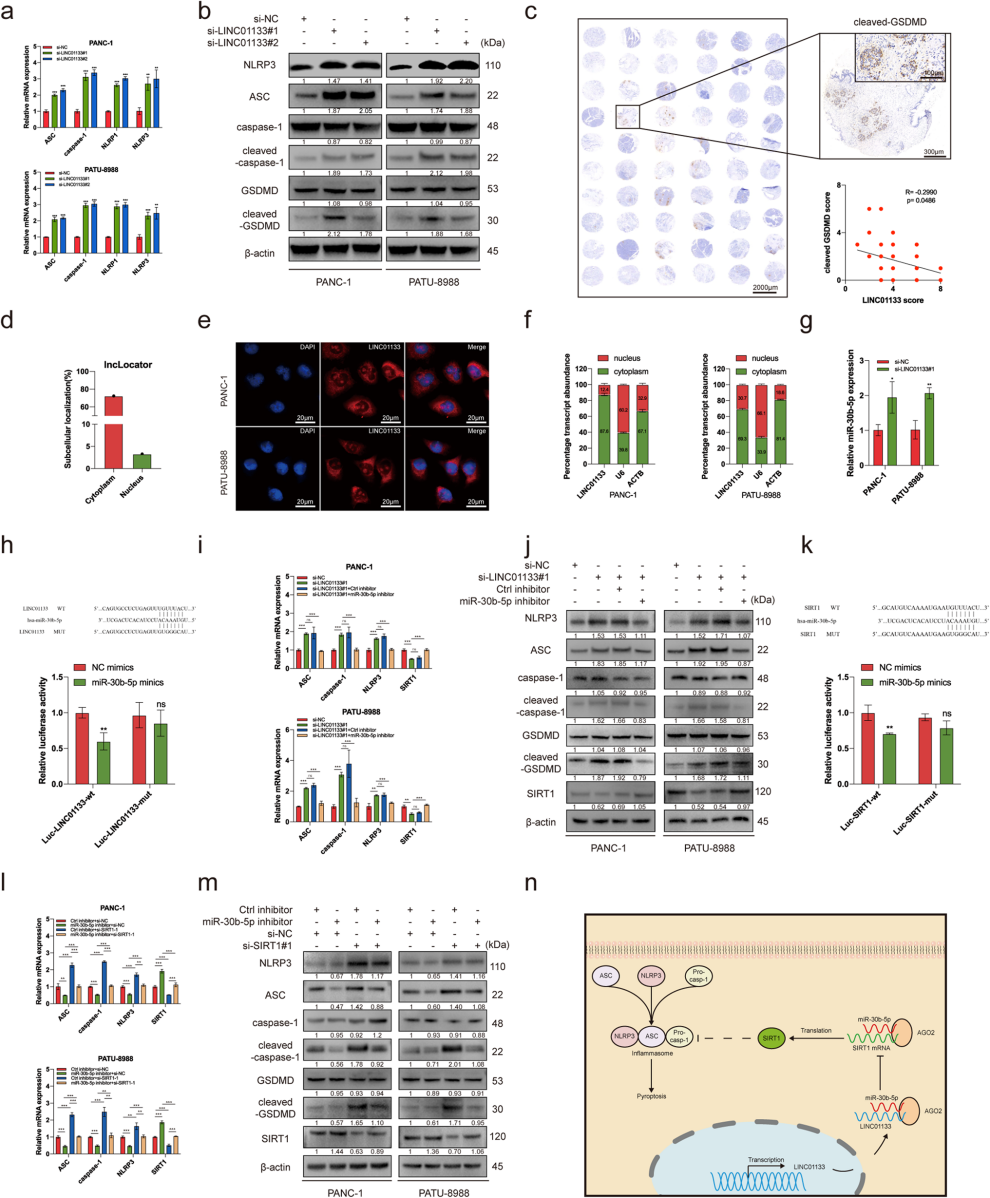
LINC01133 ameliorates PAAD pyroptosis via the miR-30b-5p/SIRT1 axis. (**a, b**) Expression of pyroptosis-related genes was evaluated using qRT-PCR (**a**) and western blotting **(b)** after transfection with LINC01133 siRNAs. (**c**) Representation image of cleaved-GSDMD IHC staining in PAAD tumor tissue and adjacent tissue; the cleaved-GSDMD score was negatively correlated with the LINC01133 score. (**d**) Relative LINC01133 expression in the subcellular localization predicted by lncLocator. (**e**) Representative FISH images showing the expression of LINC01133 (red) in PANC-1 and PATU-8988 cells, and the nucleus was stained using DAPI (blue). (**f**) Relative LINC01133 expression levels in subcellular fractions. (**g**) Expression of miR-30b-5p in PAAD cells after transfection with LINC01133 siRNA. (**h**) Luciferase activity in 293 T-cells co-transfected with LINC01133 wild-type or mutant sequence and miR-30b-5p mimics. (**i, j**) Expression of pyroptosis-related genes and SIRT1 was evaluated using qRT-PCR (**i**) and western blotting (**j**) with indicated treatments. (**k**) Luciferase activity in 293 T-cells co-transfected with SIRT1 wild-type or mutant sequence and miR-30b-5p mimics. (**l, m**) Expression of pyroptosis-related genes and SIRT1 was evaluated using qRT-PCR (**l**) and western blotting (**m**) with indicated treatments. (**n**) Proposed model demonstrating how LINC01133 inhibits pyroptosis via the miR-30b-5p/SIRT1 axis and promotes cell viability in PAAD cells. *p < 0.05; **p < 0.01; ***p < 0.001; ns, no significance

To further investigate the mechanism of LINC01133 in the regulation of PAAD cells pyroptosis, we performed qRT-PCR analysis and western blotting for pyroptosis markers. The mRNA expression of NLRP1, NLRP3, ASC, and caspase-1/cleaved-caspase-1 was higher in the si-LINC01133 groups than in the si-NC group (Fig. 6a). The western blotting results indicated that the levels of NLRP3, ASC, cleaved-caspase-1, and cleaved-GSDMD were elevated after transfection with LINC01133 siRNA (Fig. 6b). In addition, the IHC results implied a negative correlation of cleaved-GSDMD staining score with the LINC01133 score in patient PAAD tissues and subcutaneous tumor tissues (Fig. 5j and 6c). These results further demonstrated that LINC01133 inhibits the pyroptosis of PAAD cells.

The subcellular localization of LINC01133 was detected to explore its underlying mechanism. The online tool lncLocator indicated that LINC01133 was primarily distributed in the cytoplasm, which was then confirmed by FISH and nuclear and cytoplasmic separation assays (Fig. 6d–f). Because many lncRNAs in the cytoplasm function via the competing endogenous RNA (ceRNA) mechanism, it is reasonable to hypothesize that LINC01133 interacts directly with miRNA to exert its function. Using StarBase, miR-30b-5p was predicted as the LINC01133 target miRNA, and its upregulation at the mRNA level was verified after LINC01133 knockdown in PAAD cells (Fig. 6g). TCGA database indicated a negative correlation between LINC01133 and miR-30b-5p (See Supplementary Fig. 7a, Online Resource 2). With lower luciferase activity observed in the mimic + wt-LINC01133 group than in the NC mimic + wt-LINC01133 group, the dual-luciferase reporter assay results confirmed the direct interaction between LINC01133 and miR-30b-5p (Fig. 6h). Additionally, the transfection of miR-30b-5p inhibitor partially reversed the LINC01133 knockdown-induced upregulated expression of NLRP3, ASC, cleaved-caspase-1, and cleaved-GSDMD at the mRNA (Fig. 6i) and protein (Fig. 6j) levels. Moreover, the co-transfection of si-LINC01133#1 and miR-30b-5p inhibitor considerably rescued downregulated cell viability compared with that in the si-LINC01133#1 group (See Supplementary Fig. 7c–e, Online Resource 2). The results indicated that LINC01133 inhibits the pyroptosis of PAAD cells by functioning as a ceRNA for miR-30b-5p.

The downstream targets of miRNAs determine the function of miRNAs. Therefore, to identify the potential targets of miR-30b-5p, RAID, miRDB, and TargetScan databases were analyzed and SIRT1 was predicted as a potential target of miR-30b-5p. In TCGA database, SIRT1 expression was negatively correlated with miR-30b-5p (See Supplementary Fig. 7b, Online Resource 2). Additionally, SIRT1 expression at the mRNA (Fig. 6i, l) and protein (Fig. 6j, m) levels decreased after transfection with si-LINC01133#1 but increased after miR-30b-5p inhibitor transfection in PAAD cells. Subsequently, the dual-luciferase reporter assay indicated a direct interaction between miR-30b-5p and SIRT1 (Fig. 6k). Knockdown of SIRT1 by siRNA upregulated the expression of pyroptosis-related genes and reversed the downregulation effect caused by miR-30b-5p inhibitor transfection (Fig. 6l, m). Moreover, low SIRT1 expression was found to inhibit PAAD cell viability and rescue the pro-effect of the miR-30b-5p inhibitor (See Supplementary Fig. 7c–e, Online Resource 2). Collectively, LINC01133 can inhibit the pyroptosis of PAAD cells and promote PAAD development via the miR-30b-5P/SIRT1 axis (Fig. 6n).

## Discussion

PAAD is a highly malignant gastrointestinal tumor. The difficulty of early diagnosis and lack of effective treatment contribute to a poor prognosis. At present, common PAAD prognostic markers, such as the clinical characteristics of patients, tumor biomarkers, and immune markers, could help predict the prognosis of patients with PAAD; however, these prognostic markers have some limitations [37]. Establishing a good prognostic prediction model and grouping patients according to their prognostic risk factors can play a key role in the treatment of pancreatic cancer.

Recently, pyroptosis has been confirmed to affect various biological activities in tumor cells, such as proliferation, migration, and drug response. Additionally, its pro-inflammatory mechanisms could change the tumor microenvironment and impact tumor immunity [38]. CD8 + T cells can inhibit tumor growth by inducing pyroptosis of tumor cells, whereas the inflammatory molecules derived from cell lysis can also affect the tumor immune microenvironment as well as the efficacy of tumor immunotherapy [17, 39]. Nanoparticle-conjugated gasdermin can induce the pyroptosis of 15% of breast cancer cells, which can enhance the antitumor immunity effect of CD8 + T cells and finally clear the entire 4T1 mammary tumor graft [40]. Although immunotherapy has gained increasing attention and elicited encouraging responses in patients with various cancers, its effects are limited in patients with PAAD [41], possibly owing to the desmoplastic response of PAAD, lack of active immune cells, and unique tumor microenvironment of PAAD [42]. Therefore, combining the targeting of key molecules that regulate the pyroptosis of tumor cells with checkpoint blockades is of great importance for antitumor therapy. Pyroptosis‑related genes such as NLRP1 and CASP4 can regulate the proliferation and invasion of PAAD cells and serve as a prognostic signature to model patient survival [43]. However, limited studies have reported the function and prediction value of pyroptosis-related lncRNAs in PAAD.

In this study, first, we identified a pyroptosis-related prognostic signature comprising seven lncRNAs using TCGA and validated the signature in an independent cohort. Our PRL signature had a significantly higher AUC value of the ROC than other clinical features (age, sex, grade, AJCC stage, T stage, and N stage) and showed a better predictive ability for prognosis in patients with PAAD. Second, our prognostic model could provide patients with PAAD with more useful information regarding their decision on an effective treatment plan. A previous study showed that immunotherapy might be more beneficial for patients with higher TMB scores [44]. In our study, the patients in the high-risk subgroup with a greater immunosuppressive state and a higher TMB score might benefit more from immunotherapy [45]. Moreover, anti-CTLA-4 or/and anti-LAG-3 immunotherapy might lead to better outcomes for patients in the low-risk subgroup as these patients have increased expression of CTLA-4 and LAG-3. Additionally, paclitaxel and docetaxel might have greater efficacy in patients in the high-risk subgroup as these patients have lower IC50 values for these drugs. Third, we identified a novel hub lncRNA LINC01133, which exerts an anti-pyroptosis effect to promote PAAD development, and determined its functional mechanism for regulating pyroptosis.

LINC01133 was demonstrated to play a role in PAAD cell proliferation and migration. Periostin-induced LINC01133 interacts with EZH2 to regulate the Wnt/β-catenin pathway and promote the epithelial to mesenchymal transition of PAAD cells [25]. Additionally, C/EBPβ-induced LINC01133 activates cyclin G1 expression to promote PAAD proliferation [26]. However, little is known about the relationship between LINC01133 and pyroptosis. Herein, we found that LINC01133 was upregulated in PAAD tissues and associated with poor prognosis in patients with PAAD. CCK8 assay, TUNEL assays, FACs results, and in vivo experiments verified its function to promote PAAD cell viability. Moreover, the qPCR and western blotting results of pyroptosis-related genes further indicated that LINC01133 suppressed pyroptosis to promote PAAD cell viability. The IHC and ISH results suggested that the expression of cleaved-GSDMD, a key effector of pyroptosis, was negatively correlated with LINC01133 expression in patients with PAAD and xenograft PAAD tissues. Collectively, these results suggested that LINC01133 could act as a potential biomarker in PAAD and revealed a novel mechanism as LINC01133 that could promote PAAD development by inhibiting pyroptosis.

Because LINC01133 was mainly located in the cytoplasm, we speculated that it functions via the ceRNA mechanism. The direct interaction between LINC01133 and miR-30b-5p was predicted by bioinformatics analysis and validated by dual luciferase reporter assays. miR-30b-5p acts as a tumor-suppressing miRNA in papillary thyroid carcinoma [46], esophageal squamous cell carcinoma [47], and lung cancer by affecting various tumor cell bioactivities, such as proliferation, migration, and drug resistance [48]. In PAAD, miR-30b-5p promotes angiogenesis by downregulating GJA1 expression [49]. In addition, miR-30b-5p promotes cell pyroptosis via the PIK3R2/Akt/mTOR pathway in glutamic acid-induced interstitial cells [50]. However, little is known about the role of miR-30b-5p in PAAD cells. Here, the expression of miR-30b-5p was negatively correlated with LINC01133 and downregulated after LINC01133 knockdown. Transfection with miR-30b-5p partially reversed LINC01133 knockdown-mediated PAAD cell pyroptosis. This study indicated, for the first time, that LINC01133 could function as a ceRNA by binding to miR-30b-5p to inhibit pyroptosis.

As a histone/protein deacetylase [51], SIRT1 promotes the viability, proliferation, invasion, stemness, and chemoresistance of PAAD [52], and high levels of SIRT1 are associated with a poor prognosis in patients with PAAD [53]. In addition, SIRT1 can suppress pyroptosis in macrophages [54], cardiomyocytes [55], and vascular endothelial cells [56] via inhibits NLRP3 activation and subsequent caspase-1 cleavage and IL-1β secretion. The results of our study suggest that miR-30b-5p directly interacts with SIRT1 mRNA in PAAD cells and that LINC01133 functions as a ceRNA to “soak up” miR-30b-5p, thereby facilitating the expression of SIRT1. In turn, SIRT1 inhibits the expression of NLRP3 inflammasome-related factors and, thus, ameliorates pyroptosis and promotes the development of PAAD.

There are a few limitations to our study. First, our research was mainly focused on the analysis of bioinformatics in public databases and the sample sizes of the two databases were relatively small. Second, we did not reveal much about the detailed molecular mechanism by which SIRT1 regulates the expression of pyroptosis factors. Further studies are needed to reveal the potential mechanism of SIRT1 in PAAD cell pyroptosis.

## Conclusions

We determined the correlation between PRLs and the prognosis of patients with PAAD. The PRL signature identified in this study complements traditional clinical prognostic features and is a promising tool in the prognostic management of PAAD. Furthermore, our results suggested the regulatory role of LINC01133 and the miR-30b-5p/SIRT1 axis in PAAD pyroptosis, which indicates the potential prognostic and therapeutic value of LINC01133 in PAAD.

### Supplementary Information

Below is the link to the electronic supplementary material.

Supplementary file1 (DOCX 108 KB)

Supplementary file2 (DOCX 42996 KB)

## Data Availability

The datasets generated and/or analyzed during the current study are available in the Gene Expression Omnibus (https://www.ncbi.nlm.nih.gov/geo/) and The Cancer Genome Atlas (https://cancergenome.nih.gov/) databases. R code and local data in this article are available from the corresponding author on reasonable request.
