# Supplementary material for: A novel pyroptosis-associated lncRNA LINC01133 promotes pancreatic adenocarcinoma development via miR-30b-5p/SIRT1 axis
Source: Cell Oncol (Dordr). 2023 May 4;46(5):1381–98. doi: 10.1007/s13402-023-00818-5 (PMC10618383; doi:10.1007/s13402-023-00818-5)
Supplement: Supplementary file 1 — Supplementary file1 (DOCX 108 KB) [file 13402_2023_818_MOESM1_ESM.docx]

**Online Resource 1: Tables**

A novel pyroptosis-associated lncRNA LINC01133 promotes pancreatic adenocarcinoma development via miR-30b-5p/SIRT1 axis

*Cellular Oncology*

Jingwei Li^1,3,4,5†^, Jiewei Lin^1,2,3,4,5†^, Yuchen Ji^1,3,4,5†^, Xuelong Wang^1,3,4,5*^, Da Fu^1,3,4,5*^, Weishen Wang^1,3,4,5*^, Baiyong Shen^1,3,4,5*^

***Correspondence:**Baiyong Shen; Email: [shenby@shsmu.edu.cn](mailto:shenby@shsmu.edu.cn)

Weishen Wang; Email: [peanutswey@hotmail.com](mailto:peanutswey@hotmail.com)

Da Fu; Email: [fuda@shsmu.edu.cn](mailto:fuda@shsmu.edu.cn)

Xuelong Wang; Email: [wangxuelong100@126.com](mailto:wangxuelong100@126.com)

†Jingwei Li, Jiewei Lin, and Yuchen Ji contributed equally to this work

^1^Pancreatic Disease Center, Department of General Surgery, Ruijin Hospital, Shanghai Jiao Tong University School of Medicine, Shanghai, China

^2^Department of Thoracic Surgery, Shanghai Pulmonary Hospital, Tongji University School of Medicine, Shanghai, China

^3^Research Institute of Pancreatic Diseases, Shanghai Jiao Tong University School of Medicine, Shanghai, China

^4^State Key Laboratory of Oncogenes and Related Genes, Shanghai, China

^5^Institute of Translational Medicine, Shanghai Jiao Tong University, Shanghai, China

**Supplementary Table 1**. Pyroptosis-related gene list

| Gene |
| --- |
| AIM2 |
| BAK1 |
| BAX |
| CASP1 |
| CASP3 |
| CASP4 |
| CASP5 |
| CASP6 |
| CASP8 |
| CASP9 |
| CHMP2A |
| CHMP2B |
| CHMP3 |
| CHMP4A |
| CHMP4B |
| CHMP4C |
| CHMP6 |
| CHMP7 |
| CYCS |
| DIABLO |
| ELANE |
| GPX4 |
| GSDMA |
| GSDMB |
| GSDMC |
| GSDMD |
| GSDME |
| GZMB |
| HMGB1 |
| IL18 |
| IL1A |
| IL1B |
| IL6 |
| IRF1 |
| IRF2 |
| NLRC4 |
| NLRP1 |
| NLRP2 |
| NLRP3 |
| NLRP6 |
| NLRP7­ |
| NOD1 |
| NOD2 |
| PJVK |
| PLCG1 |
| PRKACA |
| PYCARD |
| RIPk1 |
| SCAF11 |
| TIRAP |
| TLR3 |
| TLR4 |
| TNF |
| TP53 |
| TP63 |

**Supplementary Table 2**. siRNA sequences (5′–3′) used in this study

| **Name** |  | **Sequence** |  |
| --- | --- | --- | --- |
| si-LINC01133#1 |  | AATGGATCCATTCCCTGCAACTGAA |  |
| si-LINC01133#2 |  | GAAGTGGAAGCAAAGTTCTCCAAAG |  |
| si-NC |  | UUCUCCGAACGUGUCACGUTT |  |
| si-SIRT1#1 |  | CAGGTCAAGGGATGGTATTTA |  |

**Supplementary Table 3**. miRNA mimics or inhibitor sequences (5′–3′) used in this study

| **Name** |  | **Sequence** |  |
| --- | --- | --- | --- |
| miR-30b-5p mimics |  | UGUAAACAUCCUACACUCAGCU |  |
| miR-NC |  | UUCUCCGAACGUGUCACGU |  |
| miR-30b-3p inhibitor |  | UGUAAACAUCCUACACUCAGCU |  |

­ **Supplementary Table 4**. Primer sequences (5′–3′) used in this study

|  |  | **Forward primer** |  | **Reverse primer** |
| --- | --- | --- | --- | --- |
| NLRP3 |  | GCCACGCTAATGATCGAC |  | TCTGAACCCCACTTCGG |
| CASP1 |  | CAAGTCAAGCCGCACAC |  | ACGCTGTACCCCAGATTTT |
| GSDMD |  | GTGCTTGCAGGGTGAGG |  | GCTCGTGGAACGCTTGT |
| ACTB |  | TCTCCCAAGTCCACACAGG |  | GGCACGAAGGCTCATCA |
| ASC |  | TGACGGATGAGCAGTACCA |  | GGCCTGGAGGAGCAAGT |
| LINC01133 |  | TCCCCACCAGAAAGTCC |  | CTGCAAGAGGAGAAAGCC |
| LINC02600 |  | AGTACATGGCAGAAGTGGGC |  | TGAGATACAGGCAGGTTCGC |
| AP003559.1 |  | AAGACGCAAAGACAGGGAGG |  | AACAGGGAACAAACAGGCGA |
| AP005233.2 |  | TGCTGGCATCATTCATTCTCC |  | TGGGGGAGAACATCAGAGTCA |
| AC090948.3 |  | CCTCTCCCCTCGTCAGATTG |  | CCTGGCCTTTCCACATTCATC |
| TRPC7-AS1 |  | GGACCACCTAGCAGGGACTT |  | CACGAGCCAAGTGCAGTATC |
| AC005062.1 |  | GGATGCCCAAGAAGTTCCA |  | TAGGCATCCTCCAGCTCCTT |
| SIRT1 |  | TAGCCTTGTCAGATAAGGAAGGA |  | ACAGCTTCACAGTCAACTTTGT |
| miR-30b-5p |  | ACACTCCAGCTGGGTGTAAACAT  CCTACAC |  | CTCAACTGGTGTCGTGGAGTCGG  CAATTCAGTTGAGAGCTGAGT |

**Supplementary Table 5**. FISH probe (5′–3′) used in this study

| **Name** |  | **Sequence** |  |
| --- | --- | --- | --- |
| LINC01133-FISH probe |  | CAAGAGGAGAAAGCCAGGGACT |  |
| Control-FISH probe |  | GTTCTCCTCTTTCGGTCCCTGA |  |

**Supplementary Table 6**. shRNA sequences (5′–3′) used in this study

| **Name** |  | **Sequence** |  |
| --- | --- | --- | --- |
| sh-LINC01133#1-F |  | CCGGAACCAGAAAUACUUAAUUCAACTCGAGTTGTTGTTTTCTGGTTTTTTTG |  |
| sh-LINC01133#1-R |  | AATTCAAAAAAACCAGAAAUACUUAAUUCAACTCGAGTTGTTGTTTTCTGGTT |  |
| sh-LINC01133#2-F |  | CACCAATGGATCCATTCCCTGCAACTGAACGAATTCAGTTGCAGGGAATGGATCCA |  |
| Sh-LINC01133#2-R |  | CACCGAAGTGGAAGCAAAGTTCTCCAAAGCGAACTTTGGAGAACTTTGCTTCCACTTC |  |

**Supplementary Table 7**. Correlation between PRL risk groups and clinical characteristics based on the Ruijin-PAAD cohort

| **­­Clinicopathologic** | **Case** | **PRL risk groups** | | **P-value** |
| --- | --- | --- | --- | --- |
| **parameters** | **(n=66)** | **Low** | **High** |  |
| Total | 66 | 26 | 40 |  |
| Gender |  |  |  | 0.999 |
| Male | 37 | 14 | 23 |  |
| Female | 29 | 12 | 17 |  |
| Age |  |  |  | 0.778 |
| ≥60 | 48 | 18 | 30 |  |
| < 60 | 18 | 8 | 10 |  |
| Pathological stage |  |  |  | 0.675 |
| I | 1 | 0 | 1 |  |
| II | 49 | 19 | 30 |  |
| III-IV | 16 | 7 | 9 |  |
| T stage |  |  |  | 0.999 |
| T1-2 | 2 | 1 | 1 |  |
| T3-4 | 64 | 25 | 39 |  |
| Lymph node metastasis |  |  |  | 0.515 |
| N0 | 36 | 16 | 20 |  |
| N1 | 29 | 10 | 19 |  |
| N2 | 1 | 0 | 1 |  |
| Distant metastasis |  |  |  | 0.999 |
| M0 | 63 | 25 | 38 |  |
| M1 | 3 | 1 | 2 |  |

**Supplementary Table 8**. Correlation between the LINC01133 expression level and clinical characteristics based on the Ruijin-PAAD cohort

| **­­Clinicopathologic** | **Case** | **LINC01133 expression level** | | **P-value** |
| --- | --- | --- | --- | --- |
| **parameters** | **(n=71)** | **Low** | **High** |  |
| Total | 71 | 35 | 36 |  |
| Gender |  |  |  | 0.232 |
| Male | 42 | 18 | 24 |  |
| Female | 29 | 17 | 12 |  |
| Age |  |  |  | 0.188 |
| ≥60 | 51 | 28 | 23 |  |
| < 60 | 20 | 7 | 13 |  |
| Pathological stage |  |  |  | 0.469 |
| I | 1 | 1 | 0 |  |
| II | 52 | 24 | 28 |  |
| III-IV | 18 | 10 | 8 |  |
| T stage |  |  |  | 0.999 |
| T1-2 | 2 | 1 | 1 |  |
| T3-4 | 69 | 34 | 35 |  |
| Lymph node metastasis |  |  |  | 0.623 |
| N0 | 37 | 20 | 17 |  |
| N1 | 33 | 14 | 19 |  |
| N2 | 1 | 1 | 1 |  |
| Distant metastasis |  |  |  | 0.999 |
| M0 | 66 | 33 | 33 |  |
| M1 | 5 | 2 | 3 |  |

**Supplementary Table 9**. Tissue microarray setting information

| Fig. 4h; Fig. 6c array 2 | | | | | |
| --- | --- | --- | --- | --- | --- |
| P13-T1 | P10-N | P8-T2 | P5-N | P3-T3 | P1-T1 |
| P13-T2 | P11-T1 | P8-T3 | P6-T1 | P3-N | P1-T2 |
| P13-T3 | P11-T2 | P8-N | P6-T2 | \ | P1-T3 |
| P13-N | P11-T3 | P9-T1 | P6-T3 | P4-T1 | P1-N |
| P14-T1 | P11-N | P9-T2 | P6-N | P4-T2 | P2-T1 |
| P14-T2 | P12-T1 | P9-T3 | P7-T1 | P4-T3 | P2-T2 |
| P14-T3 | P12-T2 | P9-N | P7-T2 | P4-N | P2-T3 |
| P14-N | P12-T3 | P10-T1 | P7-T3 | P5-T1 | P2-N |
| \ | P12-N | P10-T2 | P7-N | P5-T2 | P3-T1 |
| \ | \ | P10-T3 | P8-T1 | P5-T3 | P3-T2 |
| array1 |  |  |  |  |  |
| P29-T1 | P27-T1 | P25-T1 | P22-T1 | P19-T | \ |
| P29-T2 | P27-T2 | P25-T2 | P22-T2 | P19-N | \ |
| P29-T3 | P27-T3 | P25-T3 | P22-N | P20-T | P15-T |
| P29-N | P27-N | P25-N | P23-T1 | P20-N | P15-N |
| \ | P28-T1 | \ | P23-T2 | P21-T1 | P16-T |
| P30-T1 | P28-T2 | P26-T1 | P23-N | P21-T2 | P16-N |
| P30-T2 | P28-T3 | P26-T2 | P24-T1 | P21-T3 | P17-T |
| P30-T3 | P28-N | P26-T3 | P24-T2 | P21-N | P17-N |
| P30-N | \ | P26-N | P24-T3 | \ | P18-T |
| \ | \ | \ | P24-N | \ | P18-N |
| array3 |  |  |  |  |  |
| P42-T3 | P40-T1 | P38-T1 | P36-T1 | P33-T3 | P31-T1 |
| P42-N | P40-T2 | P38-T2 | P36-T2 | P33-N | P31-T2 |
| P43-T1 | P40-T3 | P38-T3 | P36-T3 | P34-T1 | P31-T3 |
| P43-T2 | P40-N | P38-N | P36-N | P34-T2 | P31-N |
| P43-T3 | P41-T1 | P39-T1 | \ | P34-T3 | P32-T1 |
| P43-N | P41-T2 | P39-T2 | P37-T1 | P34-N | P32-T2 |
| P44-T1 | P41-T3 | P39-T3 | P37-T2 | P35-T1 | P32-T3 |
| P44-T2 | P41-N | P39-N | P37-T3 | P35-T2 | P32-N |
| P44-T3 | P42-T1 | \ | P37-N | P35-T3 | P33-T1 |
| P44-N | P42-T2 | \ | \ | P35-N | P33-T2 |

T: PAAD tumor tissue; N: adjacent tissue
