# Supplementary material for: A novel pyroptosis-associated lncRNA LINC01133 promotes pancreatic adenocarcinoma development via miR-30b-5p/SIRT1 axis
Source: Cell Oncol (Dordr). 2023 May 4;46(5):1381–98. doi: 10.1007/s13402-023-00818-5 (PMC10618383; doi:10.1007/s13402-023-00818-5)
Supplement: Supplementary file 2 — Supplementary file2 (DOCX 42996 KB) [file 13402_2023_818_MOESM2_ESM.docx]

**Online Resource 2: Figures**

A novel pyroptosis-associated lncRNA LINC01133 promotes pancreatic adenocarcinoma development via miR-30b-5p/SIRT1 axis

*Cellular Oncology*

Jingwei Li^1,3,4,5†^, Jiewei Lin^1,2,3,4,5†^, Yuchen Ji^1,3,4,5†^, Xuelong Wang^1,3,4,5*^, Da Fu^1,3,4,5*^, Weishen Wang^1,3,4,5*^, Baiyong Shen^1,3,4,5*^

***Correspondence:**Baiyong Shen; Email: [shenby@shsmu.edu.cn](mailto:shenby@shsmu.edu.cn)

Weishen Wang; Email: [peanutswey@hotmail.com](mailto:peanutswey@hotmail.com)

Da Fu; Email: [fuda@shsmu.edu.cn](mailto:fuda@shsmu.edu.cn)

Xuelong Wang; Email: [wangxuelong100@126.com](mailto:wangxuelong100@126.com)

†Jingwei Li, Jiewei Lin, and Yuchen Ji contributed equally to this work

^1^Pancreatic Disease Center, Department of General Surgery, Ruijin Hospital, Shanghai Jiao Tong University School of Medicine, Shanghai, China

^2^Department of Thoracic Surgery, Shanghai Pulmonary Hospital, Tongji University School of Medicine, Shanghai, China

^3^Research Institute of Pancreatic Diseases, Shanghai Jiao Tong University School of Medicine, Shanghai, China

^4^State Key Laboratory of Oncogenes and Related Genes, Shanghai, China

^5^Institute of Translational Medicine, Shanghai Jiao Tong University, Shanghai, China

**
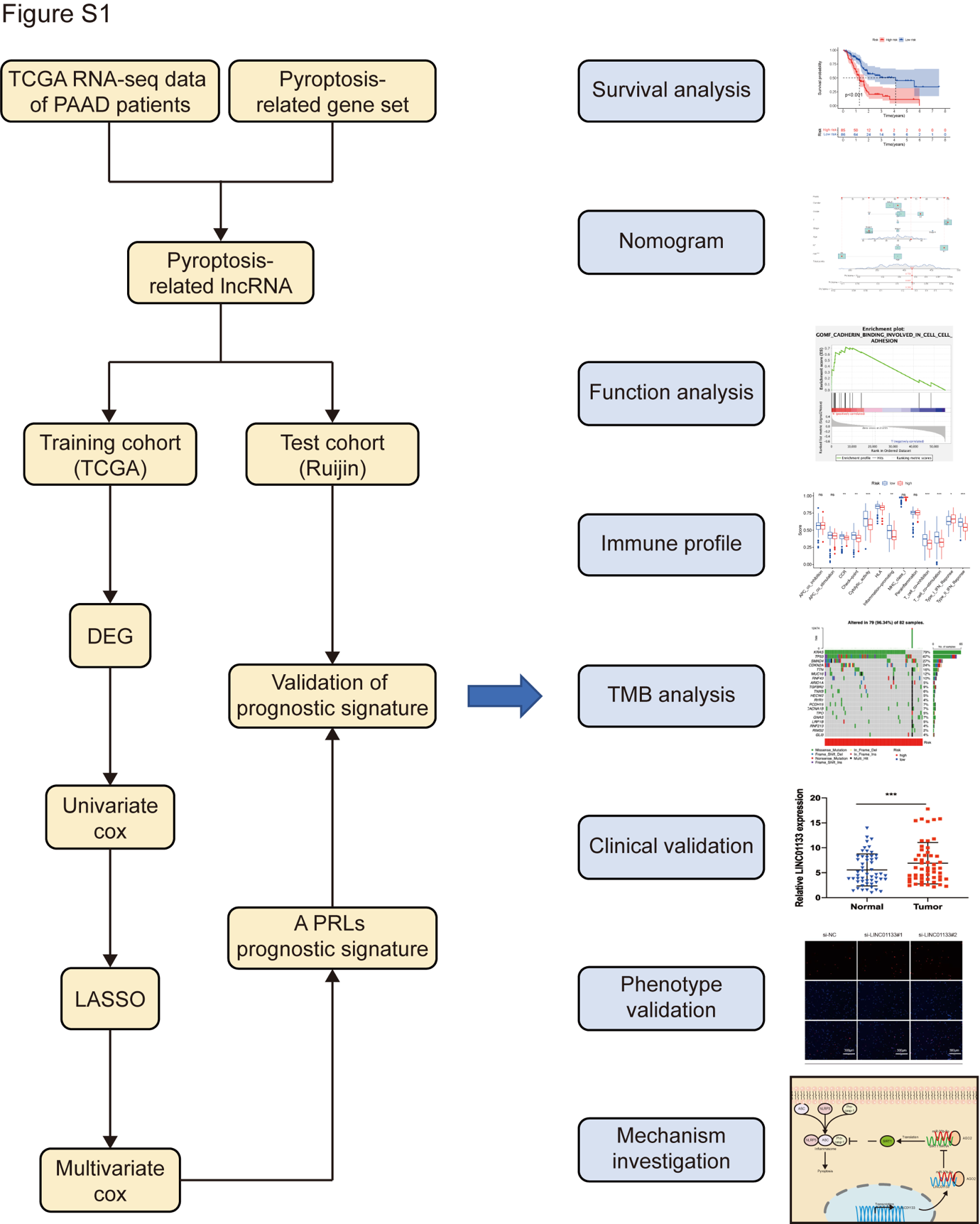
**

Supplementary Figure 1. Schematic illustration of the study methods.


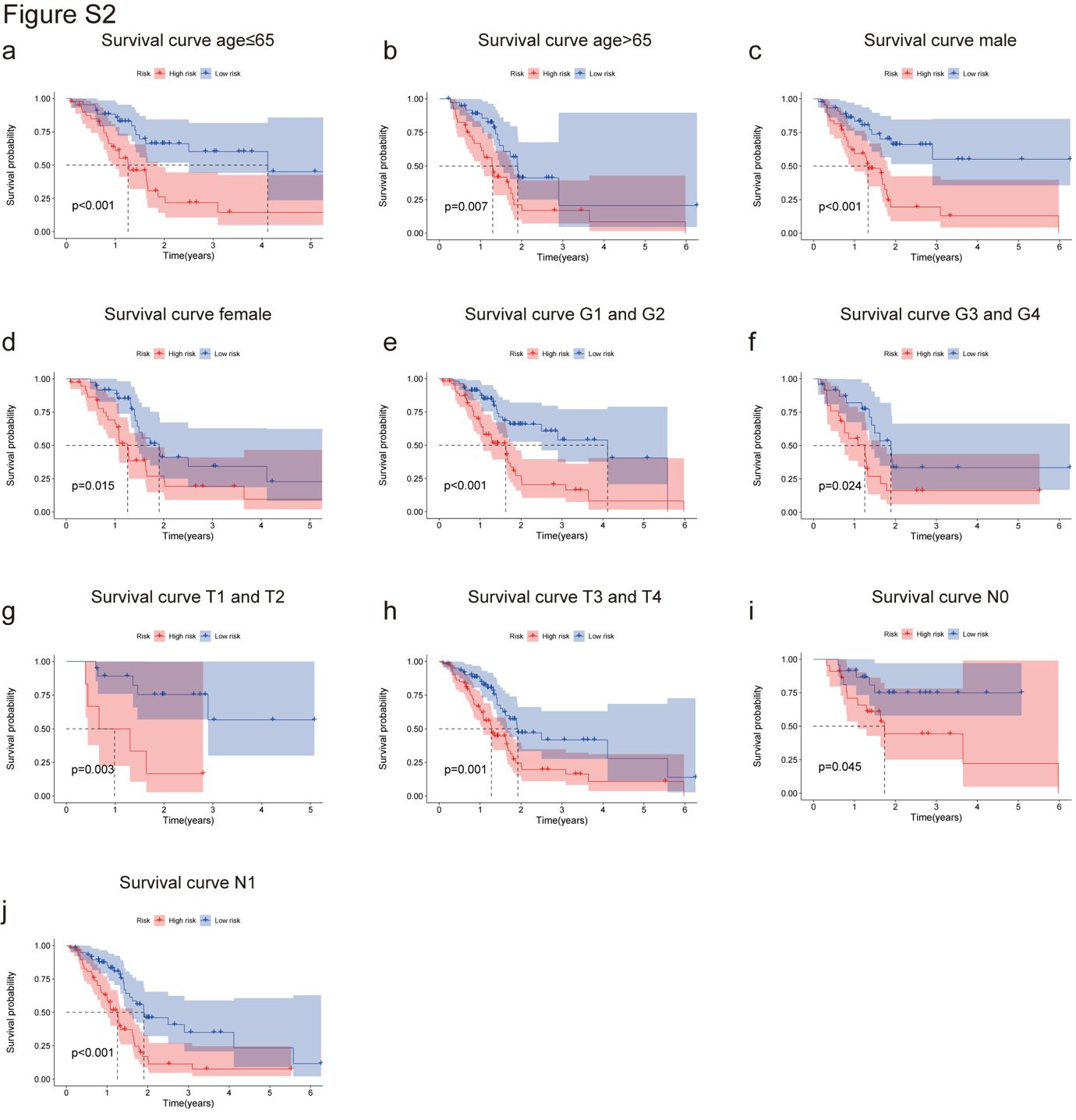


Supplementary Figure 2. Survival rates of high- and low-risk PAAD patients in the subgroups based on the clinicopathological features. **(a)** Subgroup for ages ≤65. **(b)** Subgroup for ages >65. **(c)** Male subgroup. **(d)** Female subgroup. **(e)** G1 and G2 subgroups. **(f)** G3 and G4 subgroups. **(g)** T1 and T2 subgroups. **(h)** T3 and T4 subgroups. **(i)** N0 subgroup. **(j)** N1 subgroup.


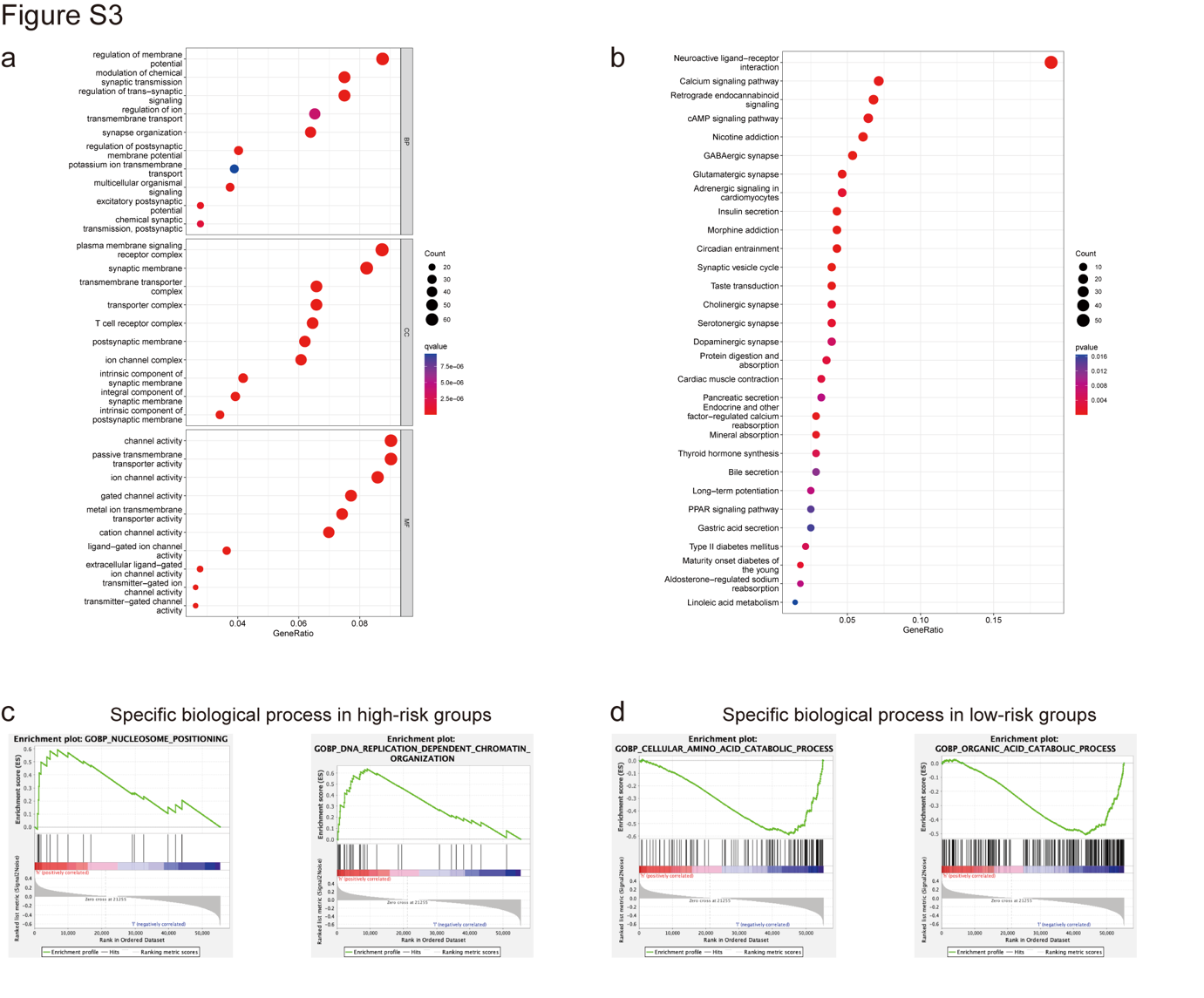


Supplementary Figure 3. Functional analysis of the PRL signature. **(a, b)** GO analysis showing the enriched biological function of DEGs between the high- and low-risk groups in TCGA. **(c, d)** GSEA of the PRL signature for GO terms.


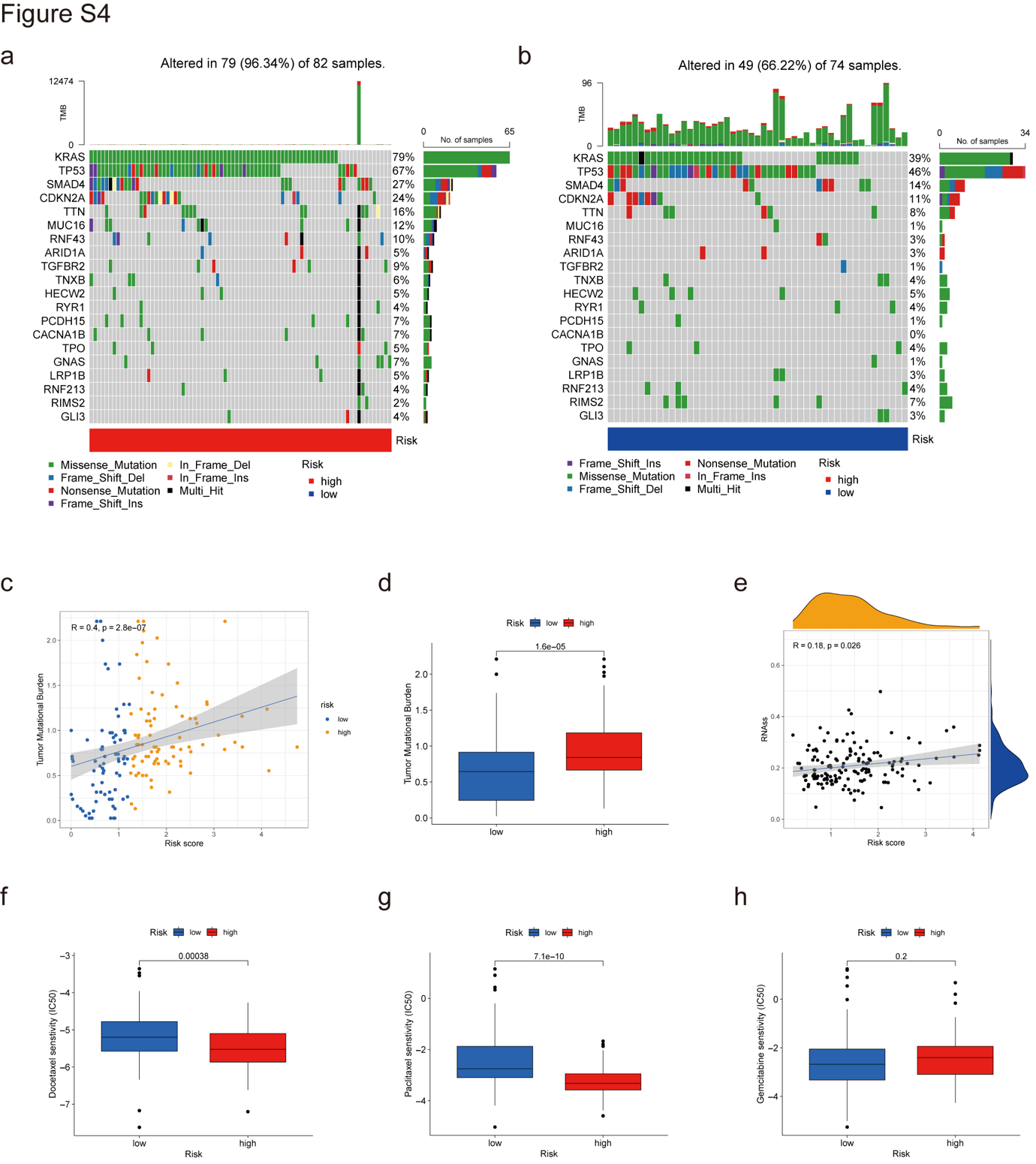


­­Supplementary Figure 4. Tumor mutational burden analysis and prediction of chemotherapy response in the two subgroups. **(a, b)** Somatic mutation landscape and TMB status in the high-risk group **(a)** and low-risk group **(b)**. **(c)** Correlation between TMB levels and the risk score. **(d)** TMB levels in the two subgroups were com­pared using *t*-test. **(e)** Correlation between cancer stem cell score and risk score. **(f–h)** Sensitivity of docetaxel **(f)**, paclitaxel **(g)**, and gemcitabine **(h)** in the two subgroups.


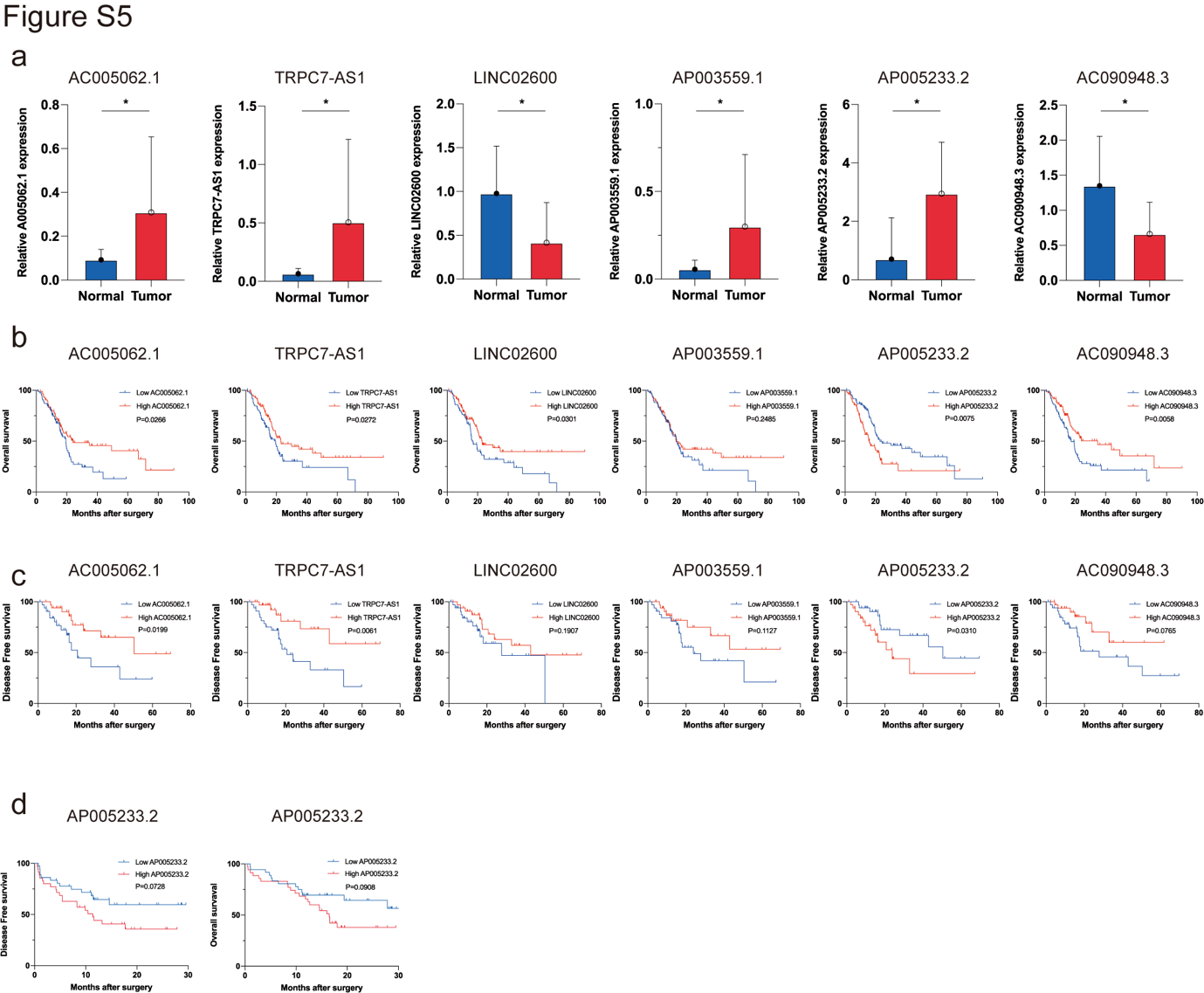
Supplementary Figure 5. Expres­sion and prognostic value of PRLs in PAAD. **(a)** Expression level of these PRLs in PAAD and normal tissues based on TCGA **(b, c)** Overall survival (OS) and disease-free survival (DFS) based on PRL expression levels were determined using TCGA database. **(d)** OS and DFS based on AP005233.2 expression levels from the Ruijin Hospital PAAD cohort.


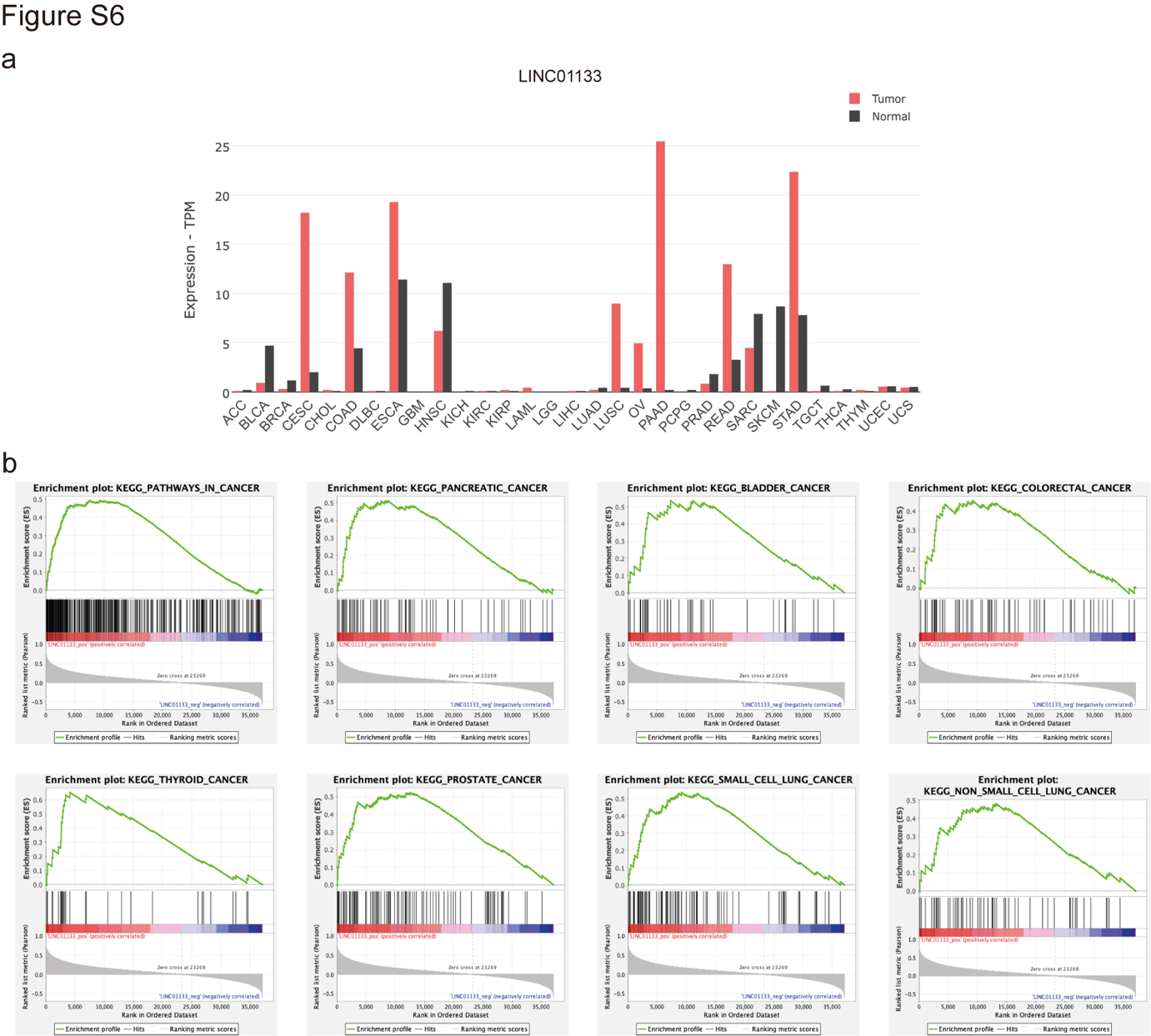


Supplementary Figure 6. Expression of LINC01133 in various types of tumors. **(a)** Expression of LINC01133 in different human tumors and normal tissues based on TCGA database. **(b)** GSEA of DEGs in the high LINC01133 expression group in TCGA database.


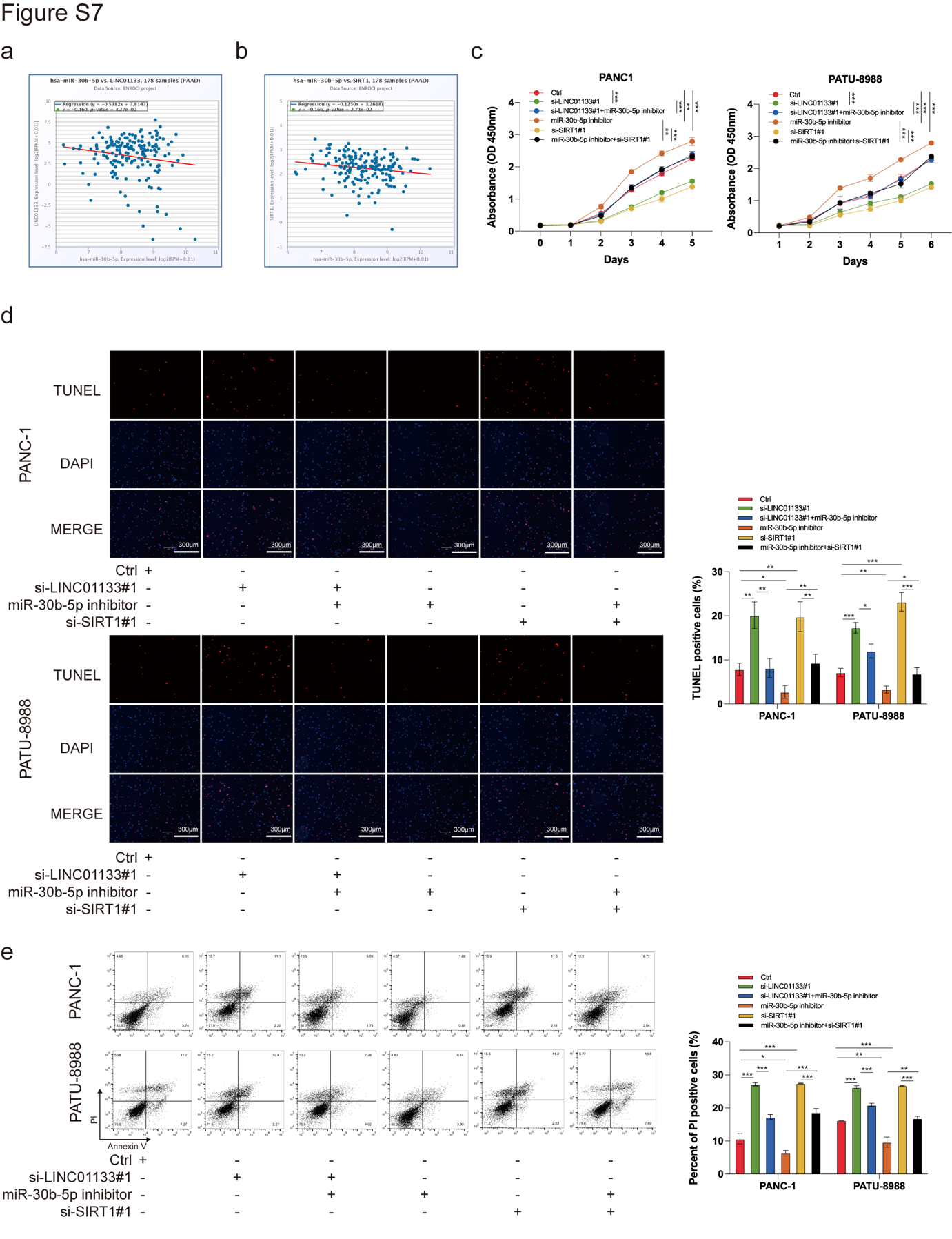


Supplementary Figure 7. MiR-30b-5p-SIRT1 axis participates in the tumor-promoting effects of LINC01133 in PAAD cells. **(a, b)** Correlation of miR-30b-5p with LINC01133 **(a)** and SIRT1 **(b)** in TCGA database. **(c–e)** CCK-8 assay **(c)**, TUNEL assay **(d)**, and cell death flow cytometry analysis **(e)** were used to detect the viabilities of PANC-1 and PATU-8988 transfected with si-LINC01133#1, miR-30b-5p inhibitor, si-SIRT1#1, or co-transfected with si-LINC01133#1 and miR-30b-5p inhibitor as well as co-transfected with miR-30b-5p inhibitor with si-SIRT1#1. *p < 0.05; **p < 0.01; ***p < 0.001; ns, no significance. All experiments were repeated three times.


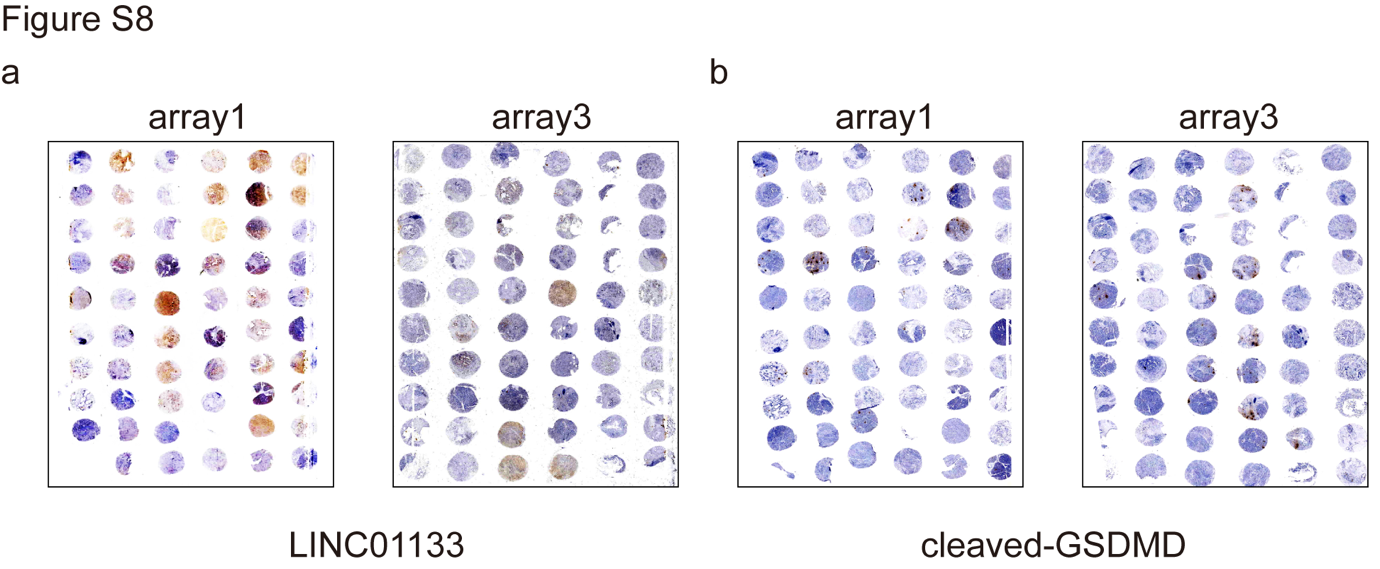


Supplementary Figure 8. IHC staining results of tissue microarrays for LINC01133 **(a)** and cleaved-GSDMD **(b)**.
